# Knockdown of CKAP2 Inhibits Proliferation, Migration, and Aggregate Formation in Aggressive Breast Cancer

**DOI:** 10.3390/cancers14153759

**Published:** 2022-08-02

**Authors:** Alexsandro dos Santos, Geneviève Ouellete, Caroline Diorio, Sabine Elowe, Francine Durocher

**Affiliations:** 1 Département de Médecine Moléculaire, Faculté de Médecine, Université Laval, Québec City, QC G1V 0A6, Canada; alexsandro.dos-santos.1@ulaval.ca (A.d.S.); genevieve.ouellette@crchudequebec.ulaval.ca (G.O.); 2 Centre de Recherche sur le Cancer, CHU de Québec-Université Laval, Québec City, QC G1V 4G2, Canada; caroline.diorio@crchudequebec.ulaval.ca; 3 PROTEO-Regroupement Québécois de Recherche sur la Fonction, L’ingénierie et les Applications des Protéines, Québec City, QC G1V 0A6, Canada; 4 Département de Médecine Sociale et Préventive, Faculté de Médecine, Université Laval, Québec City, QC G1V 0A6, Canada; 5 Département de Pédiatrie, Faculté de Médecine, Université Laval et le Centre de recherche sur le Cancer de l’Université Laval, Québec City, QC G1R 2J6, Canada

**Keywords:** breast cancer, mitosis, CKAP2, tumorigenesis, prognostic biomarker

## Abstract

**Simple Summary:**

Cancer is a complex disease where cells grow and divide in an uncontrolled manner. It is well established that its development and progression involve major alterations in the activity of mitotic regulators. In order to improve our understanding of the contribution of cell-cycle progression defects to the development of disease, the aim of this study is to identify genes relevant to the proper progression of mitosis that are deregulated in breast cancer. Our findings identified CKAP2 as an important mitotic regulator in BC tumors. Moreover, in vitro experiments showed that gene silencing of CKAP2 blocked cell growth, cell migration, and formation of cell aggregates. These results demonstrated the important role of CKAP2 in breast cancer tumor formation.

**Abstract:**

Loss of mitotic regulation is commonly observed in cancer and is a major cause of whole-chromosome aneuploidy. The identification of genes that play a role in the proper progression of mitosis can help us to understand the development and evolution of this disease. Here, we generated a list of proteins implicated in mitosis that we used to probe a patient-derived breast cancer (BC) continuum gene-expression dataset generated by our group by human transcriptome analysis of breast lesions of varying aggressiveness (from normal to invasive). We identified cytoskeleton-associated protein 2 (CKAP2) as an important mitotic regulator in invasive BC. The results showed that CKAP2 is overexpressed in invasive BC tumors when compared with normal tissues, and highly expressed in all BC subtypes. Higher expression of CKAP2 is also related to a worse prognosis in overall survival and relapse-free survival in estrogen receptor (ER)-positive and human epidermal growth factor receptor type 2 (HER2)-negative BC patients. Knockdown of CKAP2 in SKBR3 cells impaired cell proliferation and cell migration and reduced aggregate formation in a 3D culture. Our results show the important role of CKAP2 in BC tumorigenesis, and its potential utility as a prognostic marker in BC.

## 1. Introduction

Breast cancer (BC) is a highly complex and heterogeneous disease, with some cases being associated with slow growth and excellent prognosis, whilst other tumors exhibit a highly aggressive clinical course [1]. This disease ranks as the fifth leading cause of death from cancer overall, is the most frequent cause of cancer death in women in less-developed regions (14.3% of total), and is the second cause of cancer death in developed countries (15.4%) after lung cancer [2]. Recent GLOBOCAN (Global Cancer Statistics) data produced by the IARC (International Agency for Research on Cancer) from 185 countries estimated that 2.26 million women were diagnosed with BC, and 684,000 died from this disease worldwide in 2020 [3].

There are several models that explain BC progression. The linear model of disease progression states that BC progresses stepwise through different steps. It initiates as the premalignant step of atypical ductal hyperplasia (ADH), progresses into the preinvasive step of ductal carcinoma in situ (DCIS, stage 0), and culminates in the potentially lethal step of invasive ductal carcinoma (IDC, stages 1–4) [4]. In this model, ADH and DCIS are nonobligate precursors of IDC. The nonlinear (or branched) model states that DCIS is an obligatory progenitor of IDC, yet different grades of DCIS progress to corresponding grades of IDC. On the other hand, the “parallel” model of progression of DCIS and IDC hypothesizes that DCIS and IDC diverge from a common progenitor cell and progress independently through different grades in parallel. All models are strongly supported by pathologic/clinical, epidemiologic, and molecular data obtained in human BC patients as well as in animal models [5]. 

Mitosis is the evolutionarily conserved process that enables a dividing cell to equally partition its genetic material between the two daughter cells. The fidelity of mitotic division relies on the proper regulation of the expression and function of mitotic proteins. For example, spindle assembly checkpoint, kinetochore and centrosome genes are often upregulated in many cancers, including BC, and are frequently associated with genome instability [6,7], carcinogenesis [8,9] and reduced survival [10]. Patel and colleagues (2018) showed that triple negative breast cancers (TNBCs) rely on the function of specific genes within common cellular processes, such as mitosis (*BUB1*, *BUB1B*, *KIFC1*, *MASTL*, *NUF2,* and *MPS1*) and the DNA damage response (*CHEK1*, *DTL*, *RHNO1*, and *UBE2T*) [11]. Additionally, Pfister et al. (2018) found that overexpression of transcriptional regulators (such as *MYBL2*, *E2F1*, and *FOXM1*) in BC drove chromosome mis-segregation in mitosis. The authors hypothesized that dysregulation of gene expression could drive the overexpression of other mitotic regulators, which in turn lowered the robustness of mitotic pathways and, therefore, led to errors in chromosome segregation [12]. 

Loss of mitotic regulation is a common feature of cancer cells, resulting in cell-cycle dysregulation and aberrant proliferation. Because the proportion of actively dividing cells is considerably higher in cancers than in normal tissues, targeting the cell-cycle is an attractive therapeutic option for cancer treatment [13,14,15]. In BC, for example, tumor proliferation (indicated by the mitotic index) is one of the most important independent prognostic factors and is an integral part of the breast tumor grading system [16,17,18] which has also an impact on the determination of patient treatment [18,19].The identification of genes relevant to the proper progression of mitosis that are deregulated in BC can improve our understanding of the contribution of cell-cycle progression defects to the development of disease. In this study, we sought to identify mitotically relevant genes that are differentially expressed in clinically relevant steps of BC. Here, using a list of mitotic genes generated through curation of mitotically relevant GO (gene ontology) annotation terms, we queried a patient-derived BC continuum gene expression dataset generated by our group by human transcriptome analysis (HTA) of breast lesions of varying aggressiveness, namely normal, ADH (atypical ductal hyperplasia), DCIS (ductal carcinoma in situ), and IDC (invasive ductal carcinoma) [20]. From this analysis, we identified cytoskeleton-associated protein 2 (*CKAP2*) as an important mitotic regulator in invasive BC. Our results show that *CKAP2* is overexpressed in invasive BC tumors when compared with normal tissues. Moreover, *CKAP2* is highly expressed in all BC subtypes, including luminal, HER2-positive, and TNBC, when compared to normal breast tissue. Higher expression of *CKAP2* also correlates with worse relapse-free survival (RFS) and overall survival (OS) rates in ER+ and HER2-negative BC patients. Furthermore, *CKAP2* expression also positively correlates with immune-cell infiltration in BC. Finally, our results also show that knockdown regulation of *CKAP2* in the aggressive BC cell line SKBR3 impaired cell proliferation and cell migration and reduced aggregate formation in a 3D culture. Taken together, all these results show the important role of *CKAP2* in BC tumorigenesis.

## 2. Materials and Methods

### 2.1. Breast Tissue Sample Selection 

Selection of patients and data collection have been previously described [20]. Briefly, breast tissue samples were selected through a Québec-based cohort of BC patients, diagnosed pathologically, and registered at the “Centre des Maladies du Sein” (CHU de Québec). All breast tissue samples deposited were from women (53 ± 4 years) with no hormonotherapy or chemotherapy treatment before surgery. A pathologist confirmed the disease and validated clinicopathological data, such as tumor size, histologic type, grade, lymph node involvement, and receptor status, such as ER, progesterone receptor (PR), and HER2. High grade DCIS was selected to avoid any contamination with ADH and low-grade DCIS. The same was undertaken for IDC, where only high grade was selected. Normal tissue corresponded to breast tissue from patients’ routine biopsies. The protocol was approved by the Research Ethics Board of the Centre Hospitalier Universitaire de Québec, Quebec City (QC), Canada.

### 2.2. Cell Lines and Culture Conditions

The MCF10A cell line series were developed to represent different steps of BC progression. They included MCF10A (a spontaneously immortalized, non-malignant breast cell line obtained from a patient with benign fibrocystic disease) [21], MCF10AT1 (premalignant cell line derived from MCF10A overexpressing a constitutively active T24 *HRAS*) [22], MCF10DCIS.com (malignant cell line derived from a xenograft originating from premalignant MCF10AT) [23], and MCF10CA1a (invasive cell line that gained a PIK3CA H1047R activating mutation after in vivo passage of MCF10AT) [24]. MCF10A, MCF10AT1, MCF10DCIS.com, and MCF10CA1a recapitulated successive steps in BC development; namely, normal breast, ADH, DCIS, and IDC, respectively. Other IDC cell lines used in this study were MCF7, BT474, SKBR3, MDA-MB-231, and MDA-MB-468, which corresponded to different molecular subtypes of BC; namely, luminal A (lumA), luminal B (lumB), HER2, TNBC, and TNBC, respectively. 

MCF10A and AT1 cells were cultured in Dulbecco’s Modified Eagle Medium (DMEM) F-12 media (Wisent Inc., Québec, QC, Canada) supplemented with 5% horse serum (HS) (Sigma Aldrich, Oakville, CA, USA), 0.01 M HEPES (4-(2-hydroxyethyl)-1-piperazineethanesulfonic acid) (Fisher BioReagents, Ottawa Canada), 20 ng/mL epidermal growth factor (EGF) (GIBCO, Carlsbad, CA, USA), 0.01 mg/mL insulin (Wisent Inc., Québec, QC, Canada), and 500 ng/mL hydrocortisone (Sigma Aldrich, Oakville, CA, Canada). MCF10DCIS.com and CA1a were cultured in DMEM F-12 supplemented with 5% HS and 10 mM HEPES. MCF-7 cells were maintained in phenol red-free DMEM F-12 supplemented with 5% fetal bovine serum (FBS, Corning, Woodland, CA, USA), 24 mM sodium bicarbonate (Wisent Inc., Québec, QC, Canada), 0.01 M HEPES and 10 nM estradiol (E2) (Sigma Aldrich, Oakville, Canada). BT-474 cells were maintained in RPMI-1640 (Wisent Inc., Québec, QC, Canada) media supplemented with 10% FBS and 10 μg/mL insulin. SKBR3 cells were cultured in McCoy’s 5a (Wisent Inc., Québec, QC, Canada) media supplemented with 10% FBS. MDA-MB-231 and MDA-MB-468 cells were cultured in RPMI-1640 media supplemented with 10% FBS. All media were supplemented with 100 U/mL penicillin and 100 μg/mL streptomycin (HyClone™ Penicillin Streptomycin 100× Solution, Thermo Fisher Scientific).

### 2.3. RNA Extraction and Reverse Transcription-Quantitative PCR (RT-qPCR)

Total RNA from cell lines was extracted using Qiagen RNeasy mini kit (Qiagen, Hilden, Germany), according to the manufacturer’s recommendations. RT-qPCR was carried out as described previously [20]. In brief, primers were designed using GeneTools software, and their specificity was verified after blasting the GenBank database. RT-qPCR gene expression quantifications were performed and reported according to MIQE guidelines [25]. *GAPDH* and *HPRT1* were used as endogenous control. Each experiment was performed in triplicate. The primer pairs for each target gene are listed in Table S1. A melting curve analysis was carried out to assess nonspecific signals. Finally, the relative expression was subsequently calculated using the 2^−ΔΔCT^ method [26].

### 2.4. Strategy of Selecting Mitotically Relevant Genes

AmiGO (amigo.geneontology.org, accessed on 15 March 2017) is a web application that allows users to query, browse, and visualize ontologies and related gene product annotation (association) data collected from the MODs (model organism databases), UniProtKB, and other sources [27]. A list of mitotically relevant genes was generated, retrieving terms directly from this application. The terms selected were kinetochore; mitotic; centromere; centrosome; spindle; centriole; pericentriolar material; spindle pole; cytokinesis; chromosome segregation; cohesion; telomere; telomerase; transcription; translation; and replication. Table S2 lists all the annotated terms and their respective GO identifications (GO ID).

### 2.5. Identification of Differentially Expressed Genes

The normalized fold change (FC) value for each gene found on AmiGO was extracted and annotated from our previous HTA of BC samples [20]. Differentially expressed genes (DEGs) with a fold change ≥|1.5| and a *p*-value cutoff of <0.05 were defined as statistically significant. 

### 2.6. Function and Pathway Enrichment Analysis by Metascape

DEGs were analyzed using Metascape (http://metascape.org/, accessed on 21 November 2021) [28]. Pathway analysis was performed using Reactome gene sets, canonical pathways, BioCarta gene sets, GO biological processes, Hallmark gene sets, and Kyoto Encyclopedia of Genes and Genomes (KEGG); functional analysis was performed using GO molecular functions; and structural complex analysis was conducted using GO cellular components, KEGG structural complex, and CORUM (comprehensive resource of mammalian) protein complex. The 818 genes retrieved from AmiGO were used as a background dataset for the enrichment analysis. Terms with a *p*-value < 0.05, a minimum count of 3, and an enrichment factor of >1.5 were collected and grouped into clusters based on their membership similarities. The most significant term within a cluster was selected as the one representing the cluster.

### 2.7. The Analysis of Gene Expression and Prognosis from Public BC Datasets

To examine gene expression profiles, the following databases were used: Oncomine (https://www.oncomine.org/, accessed on 1 June 2021), UALCAN (http://ualcan.path.uab.edu/, accessed on 21 November 2021), GEPIA2 (http://gepia2.cancer-pku.cn, accessed on 28 October 2021), bc-GenExMiner (http://bcgenex.ico.unicancer.fr/, accessed on 10 April 2022), and TIMER2.0 (http://timer.cistrome.org/, accessed on 10 April 2022). The expression of different genes in our candidate gene list was analyzed using Oncomine [29]. Significance thresholds were set as a *p*-value less than 0.05, a fold change over 1.5, and gene rank within the top 10%. 

Gene expression profiling interactive analysis (GEPIA) was also used to compare the expression levels from The Cancer Genome Atlas (TCGA) and the Genotype-Tissue Expression (GTEx) projects. ANOVA was performed to identify the differentially expressed genes with |log2FC| values over 1 and q values less than 0.01. UALCAN (University of Alabama at Birmingham Cancer data analysis Portal) [30] was used to verify the comparison results of gene and protein expression levels and their relationship with BC classes (molecular subtypes) and individual cancer stages. Student’s *t*-test was used to generate *p*-values. *p* < 0.05 was considered to indicate a statistically significant result.

TNMplot was next used for differential gene expression analysis in normal tissues, tumor tissues, and metastatic tissues [31]. Gene expression included only paired tumor and adjacent normal tissues from RNAseq data (*n* = 112) and gene chip data (*n* = 70). Comparison of matched tissues with adjacent samples was done using the Wilcoxon test. The statistical significance cutoff was set at *p* < 0.05.

BC gene-expression miner (bc-GenExMiner) was used to examine annotated BC transcriptomic data (DNA microarray and RNA-seq) used to analyze prognosis based on *CKAP2* gene expression [32,33]. RNAseq data (*n* = 4421) was examined using ”targeted expression” based on different parameters, including age (≤51 and >51); nodal status (N+/N−); ER, PR, and HER2 status (ER+/ER−, PR+/PR−, HER2+/HER2−); molecular subtypes; and triple-negative status (TNBC vs. Not TNBC). To evaluate the difference of gene expression among the mean of different subgroups, Welch’s *t*-test was used. *p* < 0.05 was considered to indicate a statistically significant result.

Correlation among gene expression, somatic copy number alterations (CNA), and immune infiltration was calculated in BC using Tumor Immune Estimation Resource (TIMER) 2.0 [34]. For gene expression, the Wilcoxon test was computed. For immune infiltration, purity adjustment was selected using Spearman’s correlation. Results were considered statistically significant when *p*-value < 0.05. 

Prognostic significance was evaluated in Prediction of Clinical Outcomes from Genomic Profiles (PRECOG) [35] using meta-z-scores, which consists of meta-analysis of z-scores derived from individual studies for each gene in each cancer type. A meta-z-score < 1 indicates a positive association and a meta-z-score > 1 indicates a negative association with survival.

Gene expression correlation was analyzed from overall survival (OS) and relapse-free survival (RFS) in BC patients determined from the Kaplan–Meier Plotter (Kmplot) [36]. Patients were split into two groups (high and low expression) using the median of gene expression level, and only the JetSet best probe set was selected for this analysis. Then the two cohorts of patients were compared, and the univariate Cox regression was performed to calculate the hazard ratio (HR) with 95% confidence intervals (CIs) and log rank *p*-value.

### 2.8. Identification of CKAP2 Co-Expressed Genes from TCGA Datasets

For this analysis, the open-source software platform cBioPortal for Cancer Genomics (http://www.cbioportal.org/, accessed on 26 January 2022) and TCGA were used. The TCGA-BC was chosen to extract CKAP2 co-expressed genes, and only positively correlated genes (Spearman’s correlation coefficient rs ≥ 0.5) were included. Function and pathway enrichment analysis of co-expressed genes were performed using Metascape. 

### 2.9. Western Blotting

Total cell lysates were collected in RIPA buffer [10 mM tris (pH 7.5), 150 mM NaCl, 1% NP-40, 10 mM NaF, 0.1% sodium deoxycholate, 1 mM sodium orthovanadate, 20 mM b-glycerophosphate, 10 mM sodium pyrophosphate, leupeptin (10 mg/mL), aprotinin (10 mg/mL), and 1 mM 4-(2-aminoethyl) benzenesulfonyl fluoride (AEBSF)] under agitation for 30 min at 4 °C, followed by centrifugation (20,000× *g*) for 15 min at 4 °C. The cleared supernatant was collected, and protein concentration was determined using the Pierce™ BCA (Bicinchoninic acid) Protein Assay Kit (Thermo Fisher Scientific, Waltham, MA, USA). Afterwards, 10 μg of protein were loaded onto 15% SDS-PAGE gels, then transferred onto PVDF membranes with a Trans-Blot Transfer System (BioRad, Mississauga, Canada) followed by blocking with 5% milk in TBST. Immunoblotting to evaluate the CKAP2 protein expression using CKAP2 antibody (1/10,000, rabbit polyclonal, Proteintech, Rosemont, IL, USA) was performed according to standard protocol and imaged with a Bio-Rad ChemiDoc MP.

### 2.10. Lentiviral Production and Cell Infection

In this study, pLKO.1-puro lentivirus vectors expressing three independent shRNAs against *CKAP2* (generous gift of Prof. Stéphane Gobeil from Université Laval, Québec, Canada) were analyzed. The shRNAs corresponded to: sh1, sh2, and sh3. A vector expressing anon-specific sequence (labeled as scrambled or scr) was used as the control. Briefly, for lentivirus production, 4 × 10^6^ of HEK-293-T cells were plated in 10 cm petri dishes. After 24 h, cells were transfected with 10.8 µg psPAX2 (packaging plasmid), 1.2 µg pMD2.G (envelope plasmid), 12 µg of pLKO.1-puro lentivirus vector plasmids (scr or shRNAs), and 120 µg of PEI together with culture media for 16 h. Subsequently, transfection media were changed to fresh media and the virus-containing media were collected after 48 h (virus soup). The virus soup was filtered through a 0.45 µm filter to remove any residual HEK-293-T cells and stored at −80 °C for subsequent experiments. To infect SKBR3 cells and generate cell lines stably depleted of *CKAP2*, 2 mL of virus soup was mixed with fresh medium containing 10 µg/mL polybrene and incubated at 37 °C in 5% CO_2_ for 16 h. A plate with non-infected cells was kept in parallel as the control for cell death after antibiotic selection. Media were replaced with fresh media containing 1 µg/mL of puromycin, and selection was carried out for 5 days (until all non-infected cells were dead). Pools of resistant cells were collected and passaged in standard growth media. Western blot was performed to verify the efficiency of CKAP2 knockdown. 

### 2.11. Cell Growth Assays

Stably transduced SKBR3 cell line expressing shRNA against *CKAP2* (sh1, sh2 and sh3) and scrambled vector were seeded in duplicate onto 35 mm plates (25,000 cells per plate), with a culture medium change every two days. After their attachment on the plate, at days 1, 3, 5, and 7, cells were rinsed with cold PBS and trypsinized. Once trypsin was neutralized, cells were gently mixed, fixed in 1 mL final volume of 3.7% formaldehyde, and counted on the hemocytometer. 

### 2.12. Wound Healing Assay 

Cell migration was evaluated using the IBIDI culture insert (2 × 0.22 cm^2^; Ibidi, Martinsried, Germany) according to the manufacturer´s instructions. Briefly, the inserts were placed in each well, then stably transduced SKBR3 cell lines expressing shRNA against CKAP2 (sh1, sh2 and sh3) and scrambled vector were seeded (in triplicates) into each of the two insert chambers (25,000 cells/chamber) and incubated at 37 °C in 5% CO_2_ for 24 h. The insert was then removed using a sterile tweezer, and cells were gently washed twice with 1X PBS. The well was filled with fresh media containing 5 µg/mL mitomycin-C to block cell proliferation and confirm that wound healing was completely attributed to the cell migration. Images were taken at the indicated time with an EVOS™ M5000 Imaging System (Invitrogen™, Waltham, MA, USA) and analyzed using the open-source software ImageJ (Fiji package). Each gap was divided into three fields (upper, middle, and lower) and pictures were taken for every field. To ensure that the measurements were taken at the same position, the distance between the two margins of the gap was calculated using the middle field pictures.

### 2.13. Three-Dimensional Aggregate/Spheroid Formation Assay

For aggregate/spheroid generation, stably transduced SKBR3 cell lines expressing shRNA against *CKAP2* (sh1, sh2 and sh3) and scrambled vector were seeded (in triplicate) into ultra-low-attachment (ULA) 96-well round-bottomed plates at concentrations of 5000 cells per well. Plates were incubated for up to 9 days at 37 °C in 5% CO_2_ and 95% humidity, and observed under a microscope (EVOS™ M5000 Imaging System, Invitrogen™, USA) at days 2, 5, 7, and 9. Images were analyzed using ImageJ, and volume was calculated using a specific macro developed to enable high-throughput measurements in tumor spheroids [37]. 

### 2.14. Immunofluorescence 

Cells were seeded on poly-L-lysine-coated coverslips (500 µg/mL) for 24 h, then arrested for 16 h at G2/M in media containing 4 μM RO-3306 before being released for 45 min into fresh media. After that, cells were fixed with cold methanol for 10 min at −20 °C and blocked in 3% BSA in 1X PBS for 30 min. Cells were then incubated with primary antibody for 1 h at room temperature and washed three times with 1X PBS solution. Secondary antibody incubation was performed for 1 h at room temperature before final washing and mounting on microscopy slides. Primary antibodies used for immunofluorescence were anti-centrin (1/1000 dilution, clone 20H5, mouse monoclonal, Millipore) and anti-CKAP2 (1/1000, rabbit polyclonal, Proteintech). Hoechst 33342 (Thermo Scientific) was used at 1 µg/mL.

### 2.15. Confocal Microscopy

Imaging was performed with an Olympus IX80 inverted confocal microscope equipped with a WaveFX-Borealin-SC Yokagawa spinning disc (Quorum Technologies, Guelph, Canada) and an Orca Flash4.0 camera (Hamamatsu, Hamamatsu, Japan). Images shown represent Z-projection of 20 independent acquisitions, with a distance between planes of 0.2 μm. Images shown in the same figure have been identically scaled. Image processing was performed using the plugin QuickFigures from ImageJ [38].

### 2.16. Statistical Analysis

Statistical analysis was performed with GraphPad PRISM software version 9.3.1 (San Diego, CA, USA). All assays were performed in triplicate (with the exception of growth curve, which was performed in duplicates) and repeated at least three times. Statistical analysis for the comparisons of the expression, proliferation, migration, and 3D culture were done using One-way or two-way ANOVA. *p* values smaller than 0.05 were considered significant, where * indicates *p* < 0.05, ** *p* < 0.01, *** *p* < 0.001, and **** *p* < 0.0001.

## 3. Results

### 3.1. Strategy of Selecting Relevant Mitotic Genes from a BC Continuum Dataset

**Figure 1:**
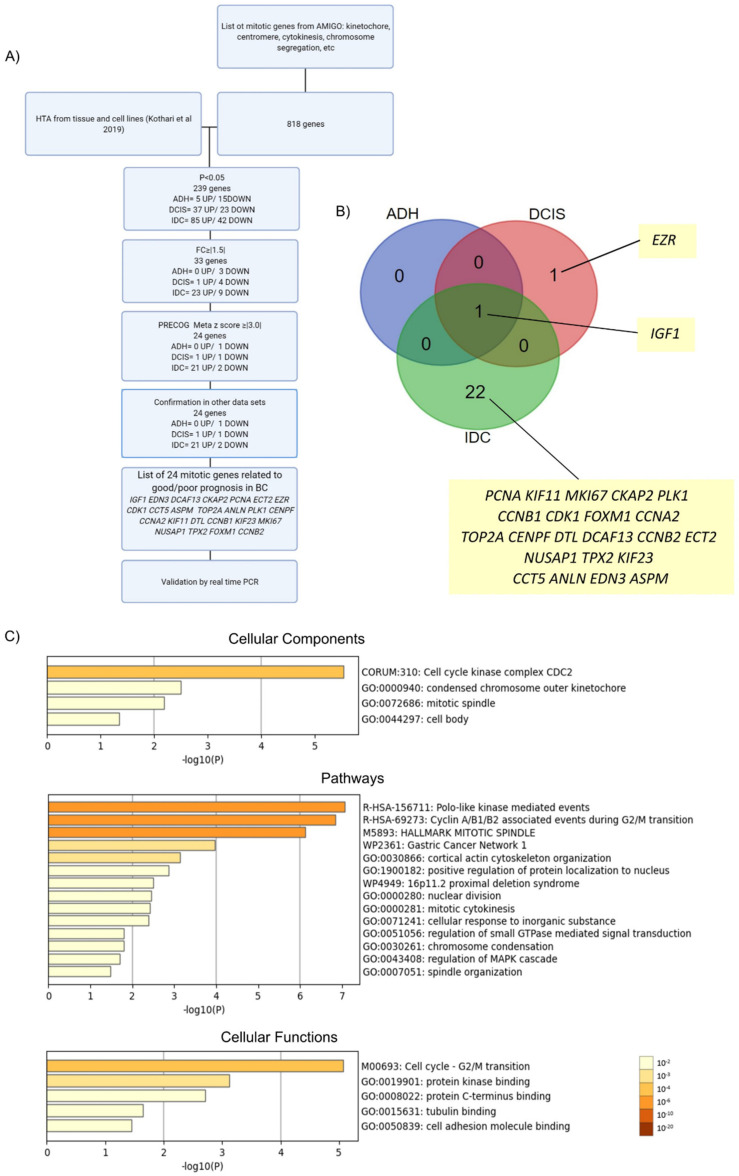
Enrichment analysis of mitotically relevant genes. (**A**) Flowchart of study design. HTA: human transcriptome array. p: p value. FC: fold change. PRECOG: Prediction of Clinical Outcomes from Genomic Profiles. (**B**) Venn diagram showing the 24 differentially expressed genes in each subgroup of BC progression compared to normal breast lesions. (**C**) Enrichment analysis of differentially expressed genes (DEGs) in BC using Metascape. Bar graph of enriched terms across input gene list, colored by *p*-values.

Previously, our group performed HTA analysis on breast tissue samples of women from the Normal, ADH, DCIS, and IDC steps, and identified a gene signature that could represent potential biomarkers for each subgroup of BC progression [20]. This work identified mitotic processes as a significantly altered pathway associated with IDC. To explore this observation further, we took a more targeted approach to identify DEGs by probing our dataset using a broad, curated list of mitotic regulators, as illustrated in the flowchart in Figure 1A. Initially, a list of mitotic genes from AmiGO was generated using specific keywords, as described in detail in the methods. This yielded a list of 818 mitotic genes, which were then used to query our recently published HTA performed on breast tissue in order to identify DEGs important for BC progression (from normal, ADH, DCIS, and IDC), as well as individual steps of the disease. To do so, FC values and *p*-values were extracted from the HTA. Using a cutoff value of *p* < 0.05, 239 unique genes were identified as significantly deregulated in expression, including 20 genes (5 upregulated and 15 downregulated) in ADH, 60 genes (37 upregulated and 23 downregulated) in DCIS, and 124 genes (85 upregulated and 42 downregulated) in IDC. An additional filtering step using absolute FC values was applied using FC ≥ |1.5| as a cutoff. This resulted in identification of 33 unique DEGs, with 3 genes (0 upregulated and 3 downregulated), 5 genes (1 upregulated and 4 downregulated), and 32 (23 upregulated and 9 downregulated) dysregulated in ADH, DCIS and IDC, respectively. 

Subsequently, PRECOG was used to evaluate the prognostic potential for BC of the 33 DEGs identified after applying the fold change cut-off. This analysis showed that 24 DEGs could be associated with survival—two genes with negative association/bad prognosis (meta z-score > −3), and 22 genes with positive association/good prognosis (meta z-score < 3) (Table 1 for meta z-scores from PRECOG). From these genes, 1 gene (0 upregulated and 1 downregulated), 2 genes (1 upregulated and 1 downregulated), and 23 (21 upregulated and 2 downregulated) were dysregulated in ADH, DCIS, and IDC, respectively (Figure 1B). Importantly, of these, one gene (*IGF1*) was found downregulated in ADH, DCIS and IDC, and was reported positively associated in the literature with BC risk [39,40], thus validating our selection strategy. The pattern of expression (up- versus down-regulation) of the above mentioned 24 genes was subsequently also further compared to publicly available BC datasets from Oncomine, UALCAN, GEPIA2, Timer2.0, and TCGA. Probing gene expression patterns validated our analysis strategy and confirmed that the expression pattern of all 24 DEGs from our HTA dataset agreed with publicly available datasets (Table 1).

Overall, using this strategy, we identified 24 mitotic genes with potential prognostic value in BC progression (Figure 1A,B and Table 1). Of these, most DEGs were upregulated, in agreement with the idea that mitotic processes are upregulated in BC [12]. These DEGs include *EZR,* which was upregulated in DCIS, and 21 genes upregulated exclusively in the IDC samples: *DCAF13*, *CKAP2*, *PCNA*, *ECT2*, *CDK1*, *CCT5*, *ASPM*, *TOP2A*, *ANLN*, *PLK1*, *CENPF*, *CCNA2*, *KIF11*, *DTL*, *CCNB1*, *KIF23*, *MKI67*, *NUSAP1*, *TPX2*, *FOXM1,* and *CCNB2*. 

To further understand the cellular components, functions, and pathways affected by the 24 DEGs, enrichment analysis was performed using Metascape. The top clusters with their representative enriched terms are shown in Figure 1C, Figure S1, and Table 2, Table 3 and Table 4. For cellular components, the DEGs were significantly enriched in cell-cycle kinase complex CDC2, condensed chromosome outer kinetochore, mitotic spindle, and cell body. Moreover, for pathways, the DEGs were mainly associated with polo-like-kinase-mediated events, cyclin-A/B1/B2-associated events during G2/M transition, and pathways implicating the mitotic spindle, including cytokinesis, spindle organization, and nuclear division. Cell-cycle G2/M transition, protein kinase binding, protein C-terminus binding, tubulin binding, and cell-adhesion molecule binding were the most enriched terms related to cellular functions. Enrichment analysis (cellular components) was also performed after every single step of filtering (*p*-value, FC, and PRECOG filters) in order to rule out bias towards the initial 818-gene list, which was also used as the background enrichment list (Figure S2). The top three GO terms for the background list were centrosome, spindle pole, and midbody. After the first filter (*p*-value < 0.05), the top three GO terms were cyclin-dependent protein kinase holoenzyme complex, kinetochore microtubule, and spindle microtubule. Following the second filter, cell-cycle kinase complex CDC2, mitotic spindle, and midbody were the top three GO terms. Finally, for the PRECOG filter, the top three GO terms were cell-cycle kinase complex CDC2, condensed chromosome outer kinetochore, and mitotic spindle. Overall, the results showed that different GO terms were found after every step of filtering when compared to the background list. Similar observations were found for pathways and cellular functions (Figure 1C) highlighting further the important role of proliferation pathways to this disease. This analysis indicates enrichment of GO annotations terms beyond the initial input dataset, and suggests that the genes, pathways, and cellular components identified in this study (Figure 1C) with roles in mitosis are significantly deregulated in BC.

### 3.2. Validation of DEGs by RT-qPCR

**Figure 2:**
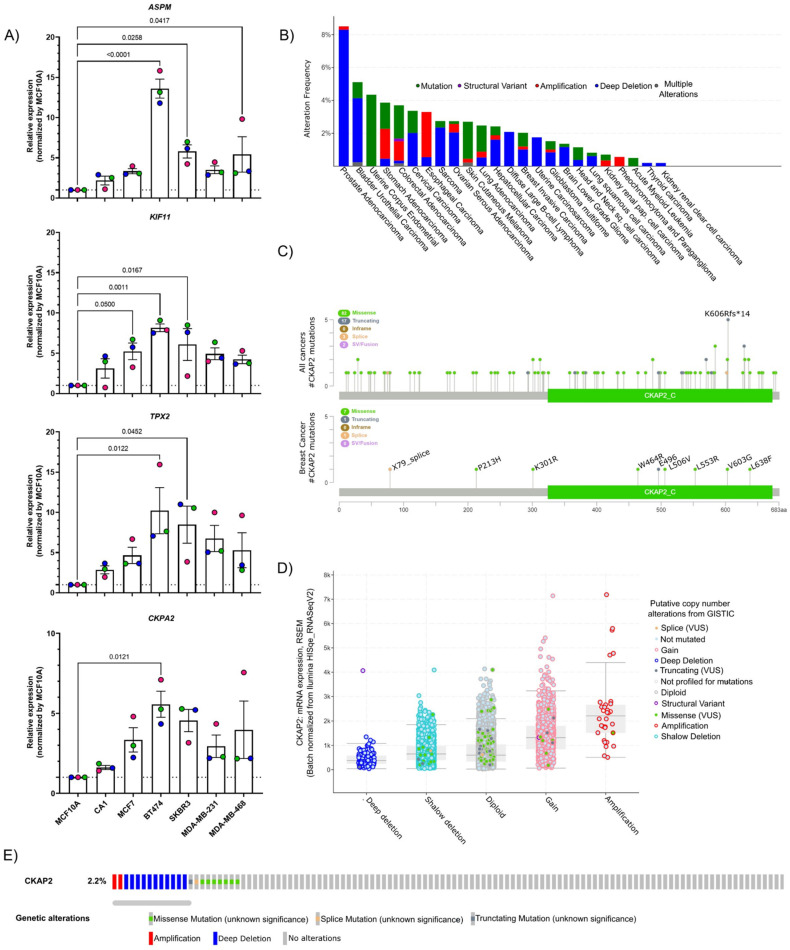
Validation of the data from HTA by qPCR and *CKAP2* mutation analysis. (**A**) Four genes were selected and validated by RT-qPCR in different breast cancer cell lines. *GAPDH* and *HPRT1* were used as the house-keeping genes to normalize mRNA-based expression data using the 2^−ΔΔCT^ method. (**B**) Bar chart of *CKAP2* mutation in pan-cancers from cBioPortal. The red bars indicate gene amplifications, blue bars are homozygous deletions, green bars are non-synonymous mutations, gray bars indicate multiple alterations. (**C**) Lollipop plot showing the distribution of *CKAP2* mutations across the coding protein from all cancers and BC. The y-axis represents the number of mutations. The x-axis represents the amino acid numbers from the domain start and stop positions. (**D**) Correlation plots illustrating the relationship between mRNA expression levels (RSEM) and putative copy number changes (Genomic Identification of Significant Targets in Cancer-GISTIC2) for the CKAP2 gene. Deep deletion, homozygously deleted; Shallow deletion, heterozygously deleted; Diploid, two alleles present; Gain, low-level gene amplification event; Amplification, high-level gene amplification event. (**E**) Oncoprint of genomic alterations found in *CKAP2* in TCGA-BC. Red bars indicate gene amplifications, blue bars are deep deletions, green bars are missense mutations, gray bars indicate truncating mutations. LumA: luminal A; LumB: luminal B, HER2+: human epidermal growth factor receptor 2 positive; TN: triple negative breast cancer; VUS: variance of unknow significance; CKAP2_C: cytoskeleton-associated protein 2 C-terminus.

To validate the HTA results using orthogonal approaches, we sought to determine expression of candidate genes in additional BC cell lines. We were particularly interested in invasive cell lines, given the number of DEGs identified in IDC (Figure 1). To this end, RNA was extracted from BC cell lines (MCF10A and CA1, which mimic the normal breast and IDC, respectively), and five other IDC cell lines corresponding to different molecular subtypes of BC, including MCF7, BT474, SKBR3, MDA-MB-231, and MDA-MB-468 (which corresponds to lumA, lumB, HER2, TNBC, and TNBC subtypes, respectively). Four DEGs, all upregulated in IDC, were selected for validation (*ASPM*, *KIF11*, *TPX2*, and *CKAP2*). *ASPM*, *KIF11,* and *TPX2* were selected because of previous reports showing them as common hits in studies of gene expression in BC cohorts, and could thus serve as further validation of our approach [12,41]. Moreover, very little is known regarding the role of *CKAP2* in BC tumorigenesis [42,43], suggesting that it might represent a promising marker or therapeutic target. Indeed, although *CKAP2* has been reported to be upregulated in various malignancies, its biological functions in the development of tumorigenesis in the breast have not been fully identified. The expression levels of all four selected DEGs were quantified using a reverse transcription-quantitative PCR (RT-qPCR) which demonstrated that the expression of *ASPM*, *KIF11*, *TPX2*, and *CKAP2* was higher in CA1 when compared to the non-tumorigenic parental cell line MCF10A (Figure 2A). Furthermore, the expression for all four genes in all IDC lines (MCF7, BT474, SKBR3, MDA-MB-231, and MDA-MB-468) was significantly higher than in MCF10A (Figure 2A). Thus, RT-qPCR results for the selected genes from independent IDC cell lines are consistent with their expression profiles obtained in the HTA dataset, and indicate that *CKAP2* is indeed upregulated in aggressive BC. 

To further explore the significance of *CKAP2* in BC, aberrations of the *CKAP2* gene were initially explored in TCGA using cBioPortal (Figure 2B). The results show that while prostate adenocarcinoma presented the highest percentage of mutations in *CKAP2* (in approximately 8% of patients), approximately 2.2% of the patients with invasive BC presented mutations in *CKAP2*. The pan-cancer analysis indicated 104 alterations in *CKAP2* protein observed in patient samples and localized throughout the protein (Figure 2C). The most common alteration was missense mutations (82 patients), followed by truncating, splicing errors, and structural variants/fusion present in seventeen, three, and two patients, respectively. Nine mutations were found in BC patients, including seven, one, and one missense, truncating, and splice variant mutations, respectively. Strikingly, most of these alterations in BC (six of nine) were found on the C terminal (CKAP2_C) domain of the protein (Figure 2C), representing the most conserved and functionally relevant region of this protein [44,45,46,47]. The pan-cancer analysis showed that the level of mRNA correlated with copy number alterations (Figure 2D), with the average expression for amplification and deep deletion presenting the highest and lowest levels of *CKAP2*, respectively. Moreover, the oncoprint showed that most alterations were missense mutations and deep deletions, although gene amplifications were also detected in BC (Figure 2E). These data taken together show the clinical relevance of *CKAP2* mutations in human cancers with the CKAP2_C domain a particular hotspot in BC.

### 3.3. Overexpression of CKAP2 in BC Tissues and in Molecular Subgroups of BC Patients

**Figure 3:**
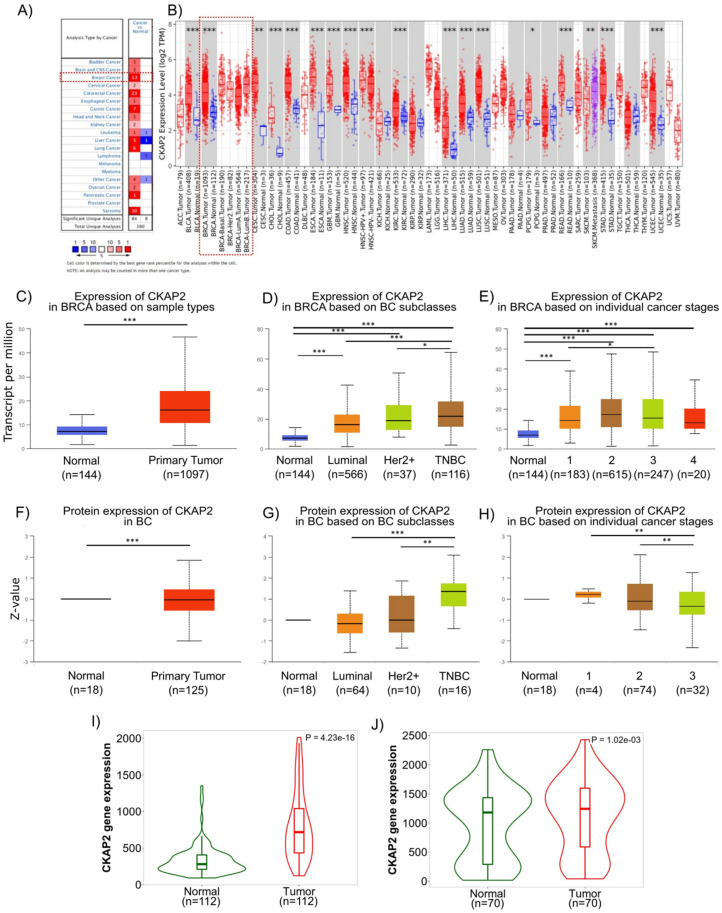
Human CKAP2 expression levels in different tumor types determined using Oncomine, Tumor Immune Estimation Resource (TIMER2.0), UALCAN (University of Alabama at Birmingham Cancer data analysis Portal), and TNMPLOT. (**A**) mRNA expression levels of CKAP2 in 20 cancer types from Oncomine. Numbers in red and blue cells represent dataset numbers in which levels of CKAP2 are statistically increased or decreased, respectively (*p* < 0.05, a fold-change > 1.5, and gene rank top 10%). (**B**) CKAP2 expression in different cancers was analyzed by TIMER2.0. (* *p* < 0.05; ** *p* < 0.01; *** *p* < 0.001). (**C**–**E**) mRNA expression of CKAP2 in BC from UALCAN. Boxplots showing CKAP2 mRNA levels in healthy controls versus individuals with BC (**C**), BC subclasses (**D**), and based on BC stages (**E**) (* *p* < 0.05; *** *p* < 0.001). (**F**–**H**) CKAP2 protein expression analysis using data from Clinical Proteomic Tumor Analysis Consortium (CPTAC). Boxplots showing CKAP2 protein levels in healthy controls versus individuals with BC (**F**), BC subclasses (**G**), and based on BC stages (**H**). (** *p* < 0.01; *** *p* < 0.001). (**I**,**J**) CKAP2 expression from paired tumor and adjacent normal tissues using TNMPLOT. (**I**) Violin plot showing CKAP2 expression from RNA-seq data. (**J**) Violin plot showing CKAP2 expression from gene chip data. BC: breast cancer; HER2+: human epidermal growth factor receptor 2 positive; TNBC: triple negative breast cancer. Data with *p* < 0.05 were considered statistically significant.

We next explored *CKAP2* gene expression in BC using publicly available databases and cancer repositories. The pan-cancer analysis of *CKAP2* expression showed that it was upregulated in several different tumor types including invasive breast carcinoma (Figure 3A,B). Two independent databases (ONCOMINE—which contained 715 cancer-related microarray datasets—and TIMER—which is based on the TCGA database) were used to evaluate the expression level of *CKAP2* across multiple cancers. Using the Oncomine, we found that *CKAP2* gene expression was upregulated when compared to normal tissue in 12 independent BC datasets (Figure 3A). Similarly, *CKAP2* gene expression was also upregulated in BC when compared to normal samples when analyzed using TIMER2.0 (Figure 3B). We next used UALCAN to further explore the correlation between *CKAP2* gene expression and protein levels in relation to molecular subtype and BC disease stage. In agreement with gene expression upregulation identified using Oncomine and TIMER2.0, data from UALCAN showed that the levels of CKAP2 mRNA were significantly higher in invasive BC tumors (*n* = 1097) than in normal tissues (*n* = 144, *p* < 0.001) (Figure 3C). In terms of stratification by BC subclasses, *CKAP2* was more highly expressed in all subclasses (luminal, HER2+ and triple negative BC–TNBC) when compared to normal breast tissue, with expression increasing as the disease progressed (Figure 3D). An analysis of the individual BC stages showed that the *CKAP2* expression was higher in all stages (stage 1–4) when compared to normal breast tissue (Figure 3E). Analysis of the CKAP2 protein levels in BC was performed using UALCAN based on the Clinical Proteomic Tumor Analysis Consortium (CPTAC) database [30,48] (Figure 3F–H). As shown in Figure 3F, protein expression levels of CKAP2 in BC were significantly increased compared to normal tissues (*p* < 0.001). Considering the different BC subclasses, the CKAP2 protein expression levels increased with increasing disease severity, essentially mimicking mRNA expression (Figure 3G). In contrast, CKAP2 protein expression appeared to change minimally with BC progression (Figure 3H). Importantly, the overexpression (at transcriptional level) of *CKAP2* was also confirmed in paired tumor and adjacent normal tissue, as evaluated using TNMplot. These results showed that the expression of *CKAP2* is higher in tumor tissue when compared to adjacent normal tissue from both RNA-seq and gene chip data (Figure 3I,J, *p* = 4.23 × 10^−16^ and *p* = 1.02 × 10^−3^, respectively). Overall, the data showed that *CKAP2* is overexpressed (at both transcriptional and protein levels) in BC tissues of different molecular subtypes, with increasing expression corresponding to increasing disease severity, in full agreement with our HTA analysis.

### 3.4. Correlation of CKAP2 Expression with Clinicopathological Parameters and Patient Survival 

**Figure 4:**
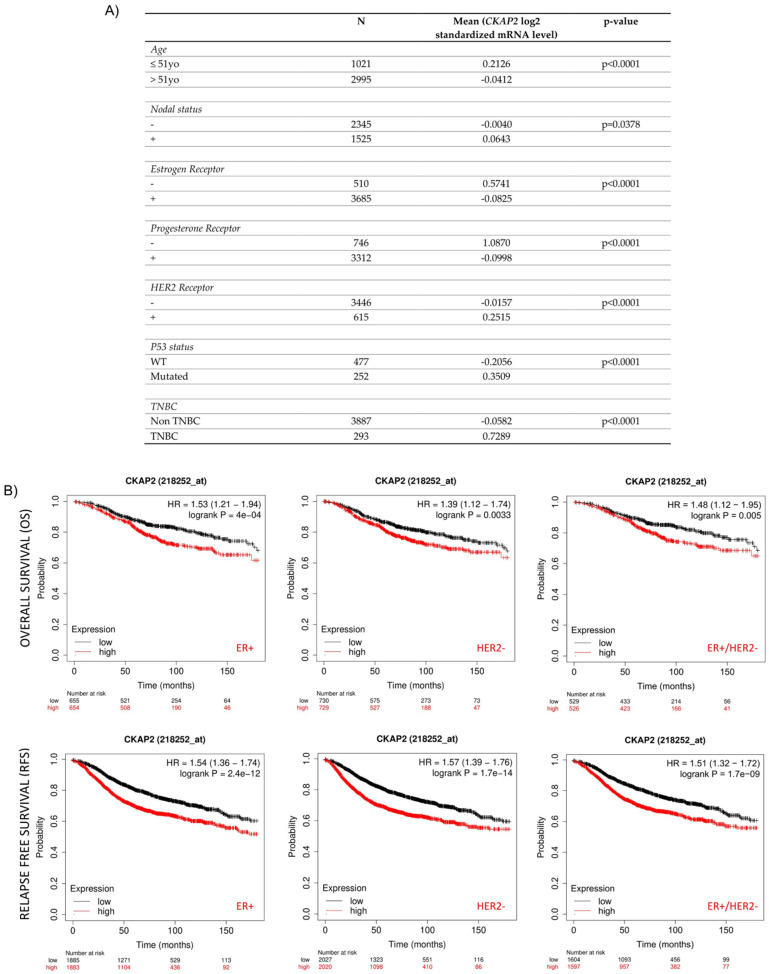
Correlation of CKAP2 expression with clinicopathological parameters and patient survival. (**A**) Relationship between CKAP2 mRNA expression and clinicopathological parameters (such as age nodal status, receptor status, p53 status, and triple-negative status) of BC generated from bc-GenExMiner. N: number of cases. (**B**) The prognostic value of CKAP2 in BC patients was plotted from KMplotter. CKAP2 expression and prognosis in BC patients were analyzed for relapse-free survival (RFS) and overall survival (OS). ER: estrogen receptor status; HER2: human epidermal growth factor receptor; HR hazard ratio. Data with *p* < 0.05 were considered statistically significant.

To compare the transcription levels of *CKAP2* between different groups of patients based on different clinicopathological indicators, and to determine the prognostic potential of *CKAP2*, the bc-GenExMiner 4.2 database was used (Figure 4A). This analysis revealed that *CKAP2* expression was higher in patients ≤51 years old when compared to patients >51 years old (*p* < 0.0001). Moreover, *CKAP2* expression was higher when nodal status was “positive” compared to when it was “negative” (*p* = 0.0378). Considering the ER and PR status, *CKAP2* expression was found to be higher when receptor was negative compared to when it was positive (ER −> ER+, *p* < 0.00001; PR −> PR+, *p* < 0.00001). Furthermore, *CKAP2* expression was higher in HER2+ when compared to HER2− (*p* < 0.0001). Additionally, *CKAP2* expression was higher when p53 was mutated compared to non-mutated (*p* < 0.0001). Lastly, *CKAP2* was highly expressed in TNBC compared to non-TNBC patients (*p* < 0.0001. These results suggest that *CKAP2* expression serves as a potential diagnostic indicator in BC, and that it may correlate with more severe disease.

The Kaplan–Meier plot was used to clarify the prognostic significance of the *CKAP2* gene in different molecular subtypes (like ER+/−, PR+/−, HER+/−, and TNBC) of BC (Figure 4B). Higher expression of *CKAP2* indicated worse overall survival (OS) in ER+ (HR = 1.53; 95% CI, 1.21–1.94; *p* = 0.0004), HER2− (HR = 1.39; 95% CI, 1.12–1.74; *p* = 0.0033), and both combined (ER+/HER2−) (HR = 1.48; 95% CI, 1.12–1.95; *p* = 0.005) patients. Moreover, higher expression of *CKAP2* indicated worse relapse-free survival (RFS) in ER+ (HR = 1.54; 95% CI, 1.36–1.74; *p* = 2.4 × 10^−12^), HER2− (HR = 1.57; 95% CI, 1.39–1.76; *p* = 1.7 × 10^−14^), and both combined (ER+/HER2−) (HR = 1.51; 95% CI, 1.32–1.72; *p* = 1.7 × 10^−9^) patients. Nevertheless, *CKAP2* expression could not predict OS and RFS in ER−, PR+/−, HER2+ and TNBC patients (data not shown). Taken together these results, which are also in line with two previous studies [42,43], showed that CKAP2 may probably have a prognostic value in OS and RFS for ER+ and HER2− patients.

### 3.5. CKAP2 Is Co-Expressed and Highly Correlated with Other Important Mitotically Relevant Genes in BC

**Figure 5:**
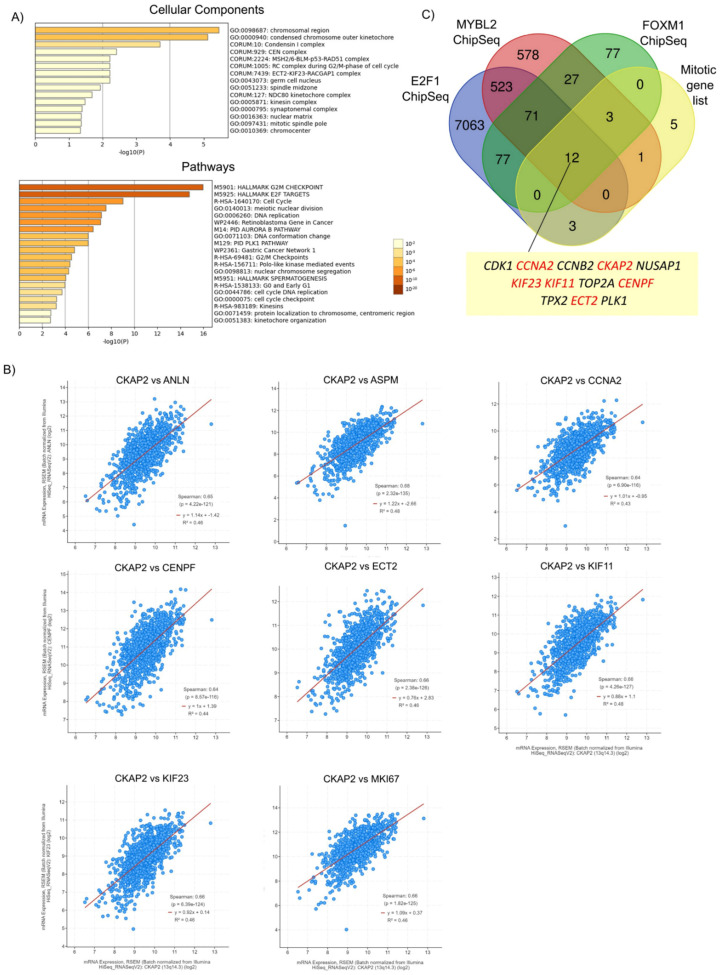
Highly correlated genes (Spearman’s correlation r ≥ 0.5) co-expressed with CKAP2 in TCGA-BRCA cohort. (**A**) Bar graph of enriched terms across input gene list, colored by *p*-values, using Metascape. Upper graph: cellular components. Lower graph: pathways. (**B**) Correlation between *CKAP2* and 8 selected genes from the top 30 highly correlated genes (r ≥ 0.5), which are also present in the list of 24 mitotically relevant genes. (**C**) Venn diagram showing the overlap of ChIP-seq datasets for *E2F1*, *MYB2L*, and *FOXM1* with the list of 24 mitotic genes. This analysis showed that all transcription factors collectively bind to the promoters of 12 mitotic genes (including *CKAP2*). Eleven genes were highly co-expressed with *CKAP2* and upregulated in IDC (highlighted in red). Data with *p* < 0.05 were considered statistically significant. *p*-value (*p*).

To further investigate the biological function of *CKAP2* and its co-expressed genes, GO enrichment analysis was performed with highly correlating genes (Spearman’s correlation r ≥ 0.5) co-expressed with *CKAP2* from TCGA-BRCA cohort using Metascape (Figure 5A and Figure S3). This analysis is important because it allows us to identify genes that are co-regulated with *CKAP2* and potentially controlled by the same transcriptional regulatory program. Here, 164 highly correlated (r > 0.5) and co-expressed genes were retrieved with *CKAP2* (Table S3). GO enrichment analysis showed that the top five enriched cellular components were related to chromosomal region, condensed chromosome outer kinetochore, condensing I complex, CEN complex, and MSH2/6-BLM-p53-RAD51 complex (Figure 5A and Figure S3). Additionally, the top five enriched pathways were mainly associated with the G2/M checkpoint, E2F targets, cell cycle, meiotic nuclear division, and DNA replication (Figure 5A). Interestingly, of the top 30 genes with the highest Spearman’s correlation coefficient (r > 0.5) co-expressed with *CKAP2*, eight (all upregulated in IDC) were present in the list of 24 mitotically relevant genes identified as deregulated in this study in IDC (Table 5 and Figure 5B). These genes were *ASPM*, *KIF11*, *ECT2*, *MKI67*, *KIF23*, *ANLN*, *CCNA2*, and *CENPF*. These data suggest that together with *CKAP2*, these may be common targets of a transcriptional program upregulated in invasive BC.

To explore this idea in more detail, publicly available datasets of chromatin immunoprecipitation followed by sequencing (ChIP-seq) were queried to determine whether known oncogenic transcription factors could bind the promoters of the genes from the list of 24 mitotically relevant genes identified in Figure 1A. *MYB2L*, *FOXM1*, and *E2F1* were selected for further analyses because previous studies found that these three transcription factors were overexpressed and highly correlated with aneuploidy status in all four BC subtypes (HER2, lumA, lumB and basal subtypes) [12]. Moreover, these transcription factors are known regulators of key proliferation programs, including those of the clusters shown in Figure 5A. For example, the functional analysis for the cluster “Polo-like kinase mediated events” showed it was highly enriched for GO terms related to “PID FOXM1 PATHWAY”, “E2F mediated regulation of DNA replication”, and “E2F-enabled inhibition of pre-replication complex formation”, which are GO terms closely related to *FOXM1* and *E2F1* transcription factors (data not shown). A Venn diagram showing the overlap among *MYB2L*, *FOXM1*, and *E2F1* ChiP-seq datasets [49,50,51] and the list of 24 mitotic genes is depicted in Figure 5C. This analysis found that the three transcription factors collectively bound to the promoters of *CKAP2* and to 11 of the 24 mitotic genes identified in Figure 1, including *CDK1*, *CCNA2*, *CCNB2*, *NUSAP1*, *KIF23*, *KIF11*, *TOP2A*, *CENPF*, *TPX2*, *ECT2,* and *PLK1*. Finally, of these 11 genes, 6 were highly co-expressed with *CKAP2* and upregulated in IDC (*CCNA2*, *KIF23*, *KIF11*, *CENPF*, and *ECT2*). Taken together, these results show that *CKAP2* overexpression in IDC may be part of a hyperactivated transcriptional program that also drives overexpression patterns of pro-mitotic genes from IDC patients. Indeed, *FOXM1* was also significantly overexpressed in our IDC samples (Figure 1B).

### 3.6. CKAP2 Expression Associates with Immune Cell Infiltration 

**Figure 6:**
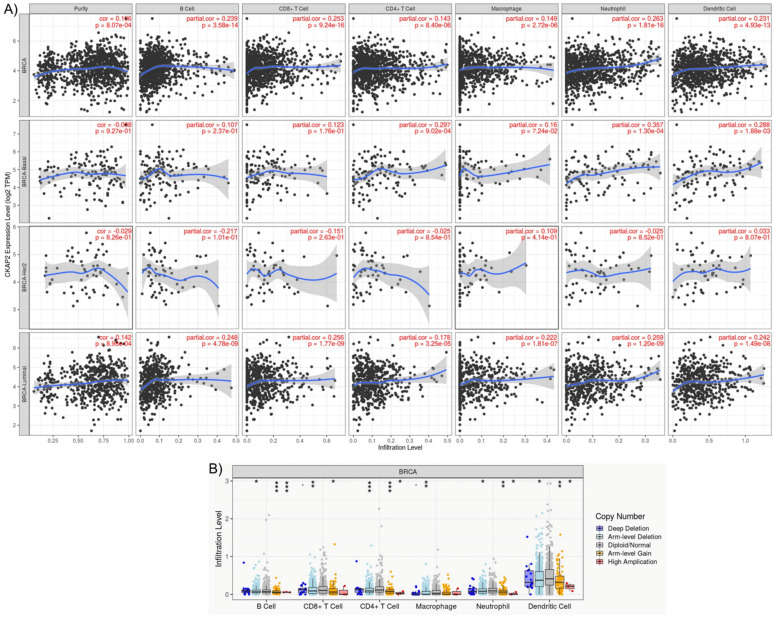
The TIMER analysis results. (**A**) Relationships between *CKAP2* expression and immune infiltration levels of B cell, CD8+ T cells, CD4+ T cells, dendritic cells, macrophages, and neutrophils in breast cancer according to TIMER2.0. (**B**) The correlation between somatic copy number alterations (SCAN) and abundance of immune infiltrates of *CKAP2*, including deep deletion, shallow deletion, diploid/normal, low-level gain, and high amplification.

The role of *CKAP2* expression and BC immunity was investigated using the TIMER2.0 database, which compiles the expression levels of tumor-infiltrating immune cells (TIICs) from TCGA cancers. This analysis is important because the BC tumor microenvironment (TME) is rich in immune infiltrates with distinct functions [52]. TIICs play essential roles in cancer development and progression, and they are an independent predictor of cancer therapy and prognosis. TIMER2.0 gives the estimation of purity (percentage of malignant cells in a tumor tissue) and abundances of six immune infiltrates (B cells, CD4+ T cells, CD8+ T cells, neutrophils, macrophages, and dendritic cells). Genes highly expressed in the TME are expected to have negative associations with tumor purity, while the opposite is expected for genes highly expressed in the tumor cells [53]. As depicted in Figure 6, *CKAP2* expression was weakly correlated with immune cell purity (*r* = 0.196, *p* = 8.07 × 10^−4^). In addition, *CKAP2* expression had small but significant positive correlations with infiltrating levels of B cells (*r* = 0.239, *p* = 3.58 × 10^−14^), CD8+ T cells (*r* = 0.253, *p* = 9.24 × 10^−4^), CD4+ T cells (*r* = 0.143, *p* = 8.40 × 10^−6^), macrophages (*r* = 0.149, *p* = 2.27 × 10^−6^), neutrophils (*r* = 0.263, *p* = 1.81 × 10^−16^), and dendritic cells (*r* = 0.231, *p* = 4.93 × 10^−13^) (Figure 6A). The correlation of *CKAP2* expression was clarified after analyzing the different subtypes of BC (Figure 6A). The results showed that luminal subtype presented small but significant positive correlations for all immune infiltrates analyzed—B cells (*r* = 0.246, *p* = 4.78 × 10^−9^), CD8+ T cells (*r* = 0.256, *p* = 1.77 × 10^−9^), CD4+ T cells (*r* = 0.17, *p* = 3.25 × 10^−5^), macrophages (*r* = 0.222, *p* = 1.81 × 10^−7^), neutrophils (*r* = 0.259, *p* = 1.20 × 10^−9^), and dendritic cells (r = 0.42, *p* = 1.49 × 10^−8^). 

Mutation of the *CKAP2* gene associated with different immune infiltrates in BC was also assessed (Figure 6B). Somatic copy number alterations were characterized by GISTIC 2.0, including deep deletion (−2), arm-level deletion (−1), diploid/normal (0), arm-level gain (1), and high amplification (2). The results demonstrated that *CKAP2* mutations (arm-level deletion, arm-level gain, and high amplification) had significant differences in B cells, CD4+Tcells, neutrophil, and dendritic cell infiltration when compared to other TIICs. These results taken together showed that *CKAP2* expression may have an important role in the immune systems of BC patients.

### 3.7. CKAP2 Expression Is High in Invasive Cell Lines and Differs between Interphasic and Mitotic Cells

**Figure 7:**
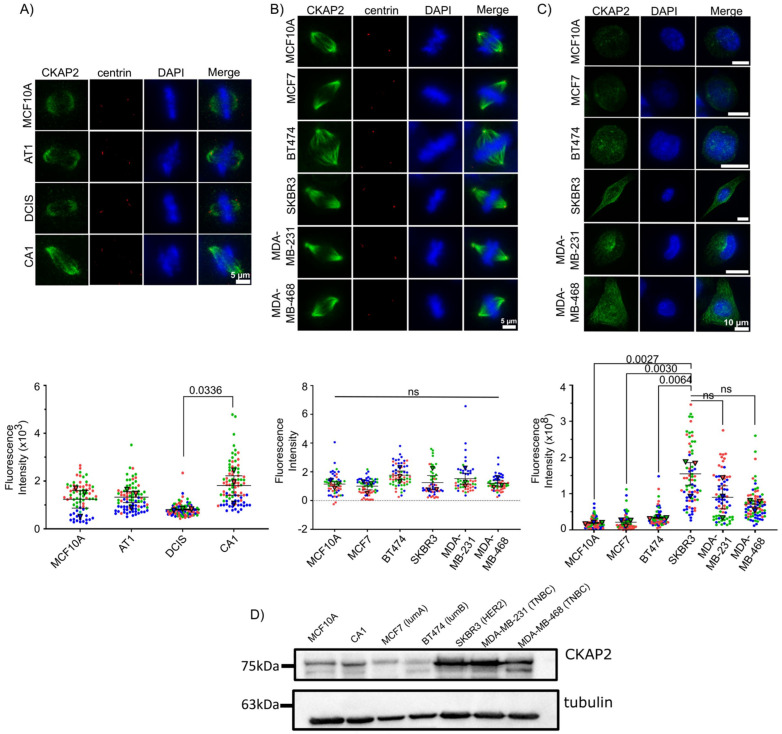
CKAP2 expression in MCF10A series and other invasive ductal carcinoma (IDC) cell lines (MCF7, BT474, SKBR3, MDA-MB-231, MDA-MB-468) by immunofluorescence. (**A**–**C**) Cells were stained with CKAP2 (green), centrin (red), and Hoechst (blue). The CKAP2 immunofluorescence intensity was measured in ≥60 cells per cell line. (**A**) Representative images of mitotic cells from MCF10A series (MCF10A, AT1, DCIS, and CA1). Superplots show the quantification of CKAP2 intensity. (**B**) Representative images of mitotic cells from other IDC cell lines (MCF7, BT474, SKBR3, MDA-MB-231, MDA-MB-468). Superplots show the quantification of CKAP2 intensity. (**C**) Representative images of interphasic cells from other IDC cell lines (MCF7, BT474, SKBR3, MDA-MB-231, MDA-MB-468). Superplots show the quantification of CKAP2 intensity. (**D**) Immunoblot showing CKAP2 protein levels in MCF10A and CA1 and other IDC cell lines (MCF7, BT474, SKBR3, MDA-MB-231, MDA-MB-468). Tubulin: loading control. LumA: luminal A, lumB: luminal B; HER2: human epidermal growth factor receptor 2 positive; TNBC: triple negative breast cancer.

In order to determine whether *CKAP2* overexpression in invasive BC can be recapitulated in another continuous model of BC, we took advantage of the MCF10A breast cancer cell line series, a powerful cell culture model system for studying BC evolution [54]. This system consists of multiple lines derived from an immortalized mammary epithelial cell line, MCF10A, propagated through sequential transplantation in mice to generate a BC continuum that mimics the way in vivo human breast lesions reflect BC progression. In this manner, these “isogenic” cell lines MCF10A, MCF10AT1, MCF10ADCIS.com and MCF10CA1a phenocopy normal, premalignant epithelium (atypical ductal hyperplasia), ductal carcinoma in situ, and high-grade invasive lesions, respectively [21,22,23,24]. To examine the levels of CKAP2, we quantified the intensity of CKAP2 levels at the spindle of mitotic cells using immunofluorescence in the MCF10A series (Figure 7A). In agreement with the HTA dataset and analysis and of publicly available BC data, our observations in the MCF10A continuum demonstrated an increase in CKAP2 levels in CA1. Additionally, data from the Human Protein Atlas (Figure S4) showed that CKAP2 staining was present in mitotic cells from IDC patients. 

CKAP2 levels were also determined in other IDC cell lines including MCF7, BT474, SKBR3, MDA-MB-231, and MDA-MB-468 (Figure 7B). Surprisingly, our results demonstrate no significant increase in CKAP2 levels at the spindle in these invasive cell lines during mitosis (Figure 7B). When CKAP2 expression was investigated in interphase, however, the results showed that the expression of CKAP2 was higher in SKBR3, MDA-MB-231, and MDA-MB-468 when compared to MCF10A (Figure 7C). These results were also reiterated in Western blots, and demonstrated again that CKAP2 was overexpressed in CA1, and to a greater extent in SKBR3, MDA-MB-231, and MDA-MB-468 interphase lysates (Figure 7D and Figure S5). Overall, CKAP2 overexpression in invasive BC was recapitulated in the MCF10A series, suggesting that it might play a role in the evolution of aneuploidy in this cell system. The surprising observation that CKAP2 overexpression was more evident in interphase rather than mitotic cells in several invasive cell lines suggests a role for this protein in regulating the interphase cytoskeleton, and supports the general conclusion that CKAP2 is expressed at higher levels in the more aggressive BC cell lines.

### 3.8. CKAP2 Knockdown Impaired SKBR3 Cell Proliferation, Migration, and Aggregate Formation In Vitro

**Figure 8:**
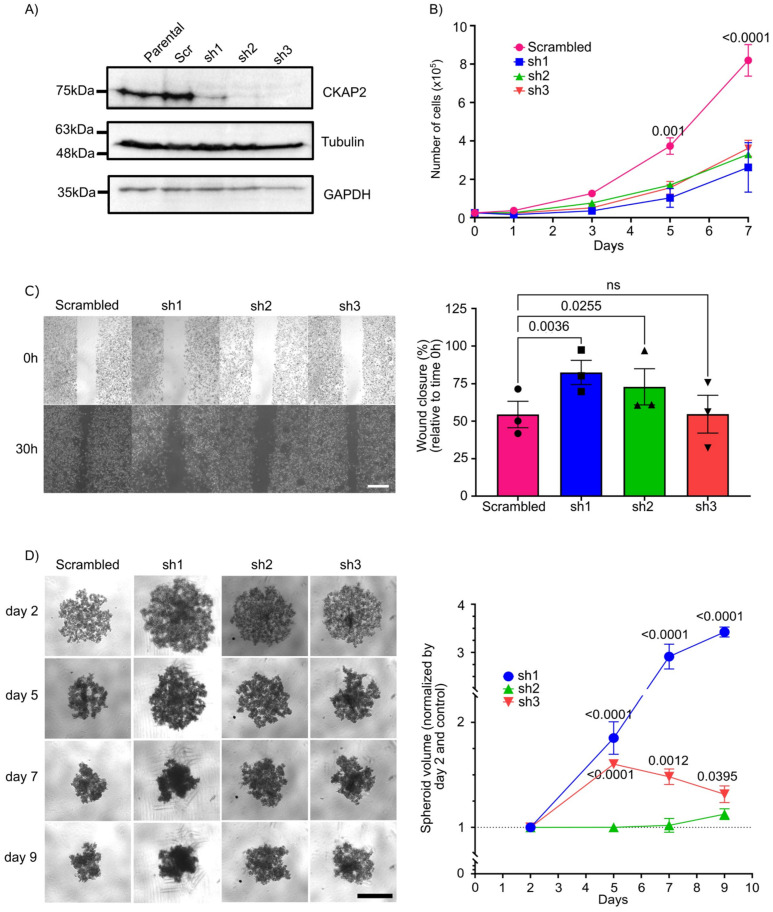
Effect of CKAP2 knockdown in breast cancer. (**A**) Immunoblot showing CKAP2 protein levels in parental SKBR3 cells and SKBR3 cells that stably express a scrambled vector (control) and three different shRNAs (sh1, sh2, and sh3). Tubulin and GAPDH: loading control. (**B**) Growth curve of CKAP2 cell lines (scrambled, sh1, sh2, and sh3). Data are shown as mean ± SEM. *p* < 0.05 were considered statistically significant. *n* = 3 biological replicates. (**C**) Cell migration (wound healing) assay in CKAP2 cell lines (scrambled, sh1, sh2, ad sh3) using IBIDI inserts. Representative images are shown from three independent experiments. Graphs show the percentage of wound closure ± SEM normalized relative to time 0. (**D**) 3D spheroid formation assay in CKAP2 cell lines (scrambled, sh1, sh2, ad sh3). Representative images are shown from three independent experiments. Graph shows the spheroid volume ± SEM normalized by control at day 2. All statistical tests of comparative data were performed using one-way (**B**,**C**) or two-way (D) ANOVA for differential comparison between more than two groups. Data with *p* < 0.05 were considered statistically significant. Scale bar: 500 µm.

To study the role of *CKAP2* in the growth, proliferation, and invasive phenotype of BC, we generated SKBR3 cell lines that stably express shRNA targeting the *CKAP2* gene. Three independent cell lines were generated targeting three different regions of *CKAP2*. Efficient knockdown of *CKAP2* was validated by Western blotting (Figure 8A and Figure S6). Using these cell lines, we first sought to validate the impact of *CKAP2* knockdown (KD) on proliferation of SKBR3 cells (Figure 8B). The growth curves shown in the graph demonstrated no differences in cell numbers between control and shRNAs KD groups at days 1 and 3. However, at later time points (days 5 and 7), we found a clear and significant decrease in the proliferation rate of *CKAP2* depleted SKBR3 cells (*p*-values for day 5 and 7 less than 0.01 and 0.0001, respectively), indicating that *CKAP2* inhibition impairs SKBR3 cell proliferation in vitro.

Cell invasion is a significant aspect of cancer progression, and involves the migration of tumor cells into contiguous tissues and the dissolution of extracellular matrix proteins [55]. We therefore sought to test the contribution of *CKAP2* to cell migration properties of SKBR3 cells using a wound healing assay (Figure 8C). Measuring closure 30 h after wound formation, in two of the three cells lines depleted for *CKAP2*, wound closure (measured as the area at t = 30 h relative to t = 0 h) was significantly enhanced (82.50% ± 5.56 relative to time 0 h, *p* = 0.0036 in sh1, and 72.92% ± 3.66 relative to time 0 h, *p* = 0.0256 in sh2) when compared to scrambled (≈50% relative to time 0h). These data indicate that the knockdown of *CKAP2* decreases the migratory potential in SKBR3 cell lines.

Three-dimensional cell cultures (including tissue explants, spheroids, and organoids techniques) have emerged as a promising method to bridge the gap between cell culture and animal models [56]. These structures phenocopy tumor-tissue-specific architecture and the pathophysiological tumor microenvironment, where tumor cells show many in vivo characteristics, such as proliferation, differentiation, motility, and metabolism [57]. In this context, we used the spheroid culture system to evaluate aspects of tumor formation upon *CKAP2* knockdown. To do so, SKBR3 cell lines expressing shRNAs against *CKAP2* or control cells were seeded in an ultra-low-attachment (ULA) plate system. SKBR3 cells transduced with scrambled vector formed aggregates in 3D culture, as expected from previous studies [58,59] (Figure 8D). We found that *CKAP2* knockdown cells (sh1 and sh3) exhibited a loosening of the formed aggregates (the volume in shRNAs transduced SKBR3 cells tended to be larger when compared to scrambled vector) at days 5, 7, and 9. The volume for sh1- and sh3-treated cells at day 5 was 1.8 (*p* < 0.0001) and 1.6 (*p* < 0.001) times larger than control cells, respectively, and this trend continued at day 7 (2.91 and 1.48 times greater when compared to control for sh1 and sh3 cells, respectively) and at day 9 (3.42 and 1.31 times greater when compared to control for sh1 and sh3 cells, respectively). Thus, the *CKAP2* knockdown reduced aggregates/spheroid formation in 3D culture when compared to the scrambled vector transduced SKBR3 cell line. Taken together, these results show that *CKAP2* knockdown impaired SKBR3 cell proliferation, migration, and aggregate formation in vitro.

## 4. Discussion

Despite the progress in the last decades in unveiling the molecular mechanisms and risk factors involved in the onset and progression of BC, and although the mortality rate has decreased in developed countries, the incidence rate has increased significantly [60,61]. Furthermore, the heterogeneous nature of this disease and the associated constellation of causative alterations complicate diagnosis, prognosis, and treatment of BC. In order to overcome this issue, and as an attempt to create more “personalized” information to guide treatment of BC patients, additional methods to classify tumors have been developed, based on single biomarkers or more complex gene signatures [20,62]. In this study, we gained insight into the mitotic gene expression profile in BC through analyzing comparative HTA performed on the different subgroups of BC progression, including Normal, ADH, DCIS, and IDC. Among the 24 DEGs identified, notable dysregulation of gene expression was observed in IDC (which presented 23 DEGs—21 of them overexpressed), including several genes previously implicated in BC, such as *IGF1*, *TOP2A*, *FOXM1,* and *TPX2*, which served to validate our approach. Our analysis resulted in the identification of CKAP2 as an important mitotic regulator in IDC. *CKAP2* gene expression is frequently upregulated in various malignancies, such as gastric cancer, ovarian cancer, glioma, and lymphoma, although little is known about its role in BC [18,45,63,64,65]. Our results from UALCAN showed *CKAP2* is overexpressed in invasive BC tumors compared with normal tissues, presenting the highest expression in HER2+ and TNBC. Bc-GenExMiner analysis showed that *CKAP2* is also highly expressed in nodal status+, ER−, PR−, HER2−, TNBC, and p53-mutated patients. Moreover, higher expression of *CKAP2* was also correlated with a worse RFS prognosis and OS in ER+ and HER2− patients.

The mitotic index and proliferation activity have been recognized as among of the most reliable breast cancer prognosticators [66,67,68]. However, there is much debate as to the reliability of routine markers, such as Ki-67, for clinical management of BC [69,70], necessitating the identification of novel biomarkers. CKAP2 is implicated in the regulation of cell division during mitosis and cytokinesis [55,63,71] and may therefore serve as a useful marker of proliferation. Indeed, Kim and colleagues demonstrated the localization of CKAP2 in the condensed chromatin of mitotic cells and the close correlation of chromatin CKAP2-positive cell count with mitotic figure count, indicating that chromatin CKAP2 could be considered as a mitosis-specific proliferation marker [18]. In agreement with this idea, prognostic significance of the CKAP2-positive cell count by immunohistochemistry in a cohort of BC patients was validated in early BC, although the prognostic significance to BC subgroups remains unclear [42,43]. Although the clinical significance of proliferation activity in the subgroups of BC patients has been not well-defined [43], the results presented here show that CKAP2 expression correlated with survival (OS and RFS) especially in HER2-negative luminal patients (ER+/HER2−), but not in HER2+ patients, which is also in agreement with Sim and colleagues [42]. The present results, in agreement with previous studies [42,43], suggest that hyperproliferation may have more impact in the ER+/HER2− subtype of BC patients. Collectively, these studies and our work confirm the importance of CKAP2 as a possible prognostic indicator in BC. Further studies are needed to understand and explore CKAP2 as a prognostic factor in subgroups of BC.

Beyond its prognostic value, we explored the functional significance of CKAP2 upregulation in BC. CKAP2 is a microtubule-associated protein that plays a role in the integrity of microtubule nucleation sites in early mitosis to accurately form the mitotic spindle and spindle poles [72]. In primary hepatocytes, for example, CKAP2 was reported to be essential for maintaining centrosome integrity and chromosome segregation [73], thereby maintaining genome stability [74]. Considering that there is very little known about CKAP2 transcriptional regulation, we tested the hypothesis that CKAP2 expression might be part of a core mitotic transcriptional program. Mining public ChiP-seq datasets, we found that the key mitotic transcription factors *MYB2L*, *FOXM1*, and *E2F1* bound to the promoter of *CKAP2* and 11 additional mitotic genes (*CDK1*, *CCNA2*, *CCNB2*, *NUSAP1*, *KIF23*, *KIF11*, *TOP2A*, *CENPF*, *TPX2*, *ECT2*, and *PLK1*), six of which were highly co-expressed with *CKAP2* and upregulated in IDC. Our work therefore suggests that *CKAP2* may be overexpressed as part of a transcriptional program deregulated in BC. Indeed *MYBL2*, *FOXM1*, and *E2F1* are drivers of aneuploidy and chromosome mis-segregation in BC [12,75,76]. Interestingly, Pfister and colleagues hypothesized that *E2F1*, *FOXM1*, and *MYBL2* overexpression lowers the fidelity of mitosis by driving the overexpression of many mitotic regulators, which thus lowers the robustness of mitotic pathways, although the exact targets involved remained unclear [12]. The authors argued that this hyperactive transcriptional program, together with the loss of TP53 function often observed in highly aneuploid breast tumors [77,78] likely generates conditions that allow highly aneuploid tumors to remain proliferative [12]. Here, we identified *CKAP2* as part of this transcriptional program. Ultimately, understanding how the fidelity of mitosis is regulated by transcriptional networks in BC will likely provide significant insight into the evolution of aneuploidy in this disease.

Despite the view that BC is a relatively non-immunogenic cancer, the BC tumor microenvironment is rich in immune infiltrates with distinct functions [52]. In addition, studies indicate that the tumor microenvironment has clinicopathological significance in predicting survival outcomes and assessing therapeutic efficacy factors [79,80]. For this reason, it is important to have a comprehensive evaluation of the immune landscape in BC and construct an immune signature related to the immune landscape. Using TIMER2.0, our results showed that *CKAP2* expression was also associated with immune cell infiltration in BC. We found that *CKAP2* expression presented weak but significant positive correlations with infiltrating levels of B cells, CD8+ T cells, CD4+ T cells, macrophages, neutrophils, and dendritic cells, indicating a potential function of *CKAP2* in regulating the tumor immunology of BC. Further studies, involving, for example, co-culture of immune cells and BC cells overexpressing (or knocking down) *CKAP2*, are needed to check whether the infiltration capacity can shed light on the influence of *CKAP2* in the immune system in BC.

To further investigate the effect of *CKAP2* on BC tumorigenesis, we investigated CKAP2 protein levels in both mitotic and interphase cells in a panel of BC cell lines and we found increased expression in aggressive cell lines in both interphase and mitosis in a cell line-dependent manner. Moreover, we created a panel of SKBR3 cell lines expressing shRNA against *CKAP2,* and successfully depleted the protein. Our results showed that knockdown of *CKAP2* in the SKBR3 cell line impaired cell proliferation and cell migration and reduced aggregate formation in a 3D culture, suggesting that this protein may be an important mediator of proliferation in aggressive BC. Although we have found that CKAP2 knockdown impairs cell proliferation, cell death cannot be discounted. Additional experiments, including TUNEL assay or flow cytometry using anexin V, for example, are needed to confirm whether CKAP2 also plays a role in cell death. To our knowledge, this is the first study to analyze in vitro the effect of *CKAP2* knockdown in BC, and provides an incentive for further mechanistic studies. Importantly, these results are in agreement with *CKAP2* studies from other types of cancer. For example, Wang and colleagues found that the silencing of *CKAP2* by siRNA suppressed the proliferative capacity and clonogenicity of glioma cells [65]. Furthermore, Guo et al. found that the downregulation of *CKAP2* by shRNA inhibited cell migration and invasion of cervical carcinoma cells in vitro and decreased the tumor growth in vivo [55]. Zhang and Zhao showed that inhibition of *CKAP2* by siRNA led to inhibition of migration in ovarian adenocarcinoma cells [63]. Additionally, Zhang and colleagues found that the *CKAP2* knockdown by shRNA impaired osteosarcoma cell growth in vivo and in vitro [81]. Taken together, all these studies show the important role of *CKAP2* in the progression of different cancers.

## 5. Conclusions

We generated a list of mitotic genes related to prognosis and, using integrative bioinformatics, identified *CKAP2* as an important mitotic regulator in IDC. *CKAP2* was overexpressed in invasive tumors, and its high expression was also correlated with worse RFS and OS in patients diagnosed with ER+ or HER2− BC. Moreover, *CKAP2* expression may also serve as a prognostic biomarker associated with immune infiltration in BC. Furthermore, our in vitro experiments showed that knockdown of *CKAP2* in the aggressive SKBR3 cell line impaired cell proliferation and cell migration and reduced aggregate formation in a 3D culture. Taken together, all these results show the important role of *CKAP2* in the BC tumorigenesis.

## Figures and Tables

**Table 1:** The 24 mitotically relevant genes related to prognosis in breast cancer identified in this study (genes highlighted in green or gray are downregulated or upregulated, respectively). ^1^ TCGA-BRCA dataset (https://www.cbioportal.org/, accessed on 26 January 2022); ^2^ UALCAN (http://ualcan.path.uab.edu/, accessed on 21 November 2021); ^3^ GEPIA (http://gepia.cancer-pku.cn/, accessed on 28 October 2021); ^4^ HTA data from Kothari and colleagues (2018); ^5^ PRECOG (https://precog.stanford.edu/, accessed on 1 June 2021); ^6^ KMplot (https://kmplot.com/, accessed on 1 October 2021). ADH: atypical ductal hyperplasia; DCIS: ductal carcinoma in situ; IDC: invasive ductal carcinoma; BLCA: bladder urothelial carcinoma; BRCA: breast invasive carcinoma; CESC: cervical squamous cell carcinoma and endocervical adenocarcinoma; LIHC: liver hepatocellular carcinoma; LUAD: lung adenocarcinoma.

	Gene Symbol	Gene Name	Expression in TCGA Cancers Other than BC ^1,2,3^	Expression in Breast Invasive Carcinoma (Compared to Normal)—TCGA ^1,2,3^	Expression (HTA Tissue Compared to Normal ^4^)	Fold Change ^4^			PRECOG ^5^	Kmplot ^6^
						ADH	DCIS	IDC		
1	*IGF1*	Insulin-like growth factor 1	underexpressed in BLCA, BRCA, CESC, LIHC, LUAD, etc.	underexpressed	underexpressed in all stages	−1.87	−2.02	−2.34	−4.7	low expression is a bad prognosis (RFS, OS and DMSF)
2	*EDN3*	Endothelin 3	underexpressed in BRCA, CESC, LUAD, etc.	underexpressed	underexpressed in IDC	−1.03	−1.23	−1.53	−3.42	low expression is a bad prognosis (RFS)
3	*DCAF13*	DDB1- and CUL4-associated factor 13	overexpressed in BLCA, BRCA, CESC, LIHC, LUAD, etc.	overexpressed	overexpressed in IDC	1.11	1.19	1.96	3.38	high expression is a bad prognosis (RFS and DMSF)
4	*CKAP2*	Cytoskeleton-associated protein 2	overexpressed in BLCA, BRCA, CESC, LIHC, LUAD, etc.	overexpressed	overexpressed in IDC	−1.09	−1.03	1.67	3.72	high expression is a bad prognosis (RFS, OS and DMSF)
5	*PCNA*	Proliferating cell nuclear antigen	overexpressed in BLCA, BRCA, CESC, LIHC, LUAD, etc.	overexpressed	overexpressed in IDC	1.02	1.09	1.6	3.91	high expression is a bad prognosis (RFS, OS and DMSF)
6	*ECT2*	Epithelial cell transforming 2	overexpressed in BLCA, BRCA, CESC, LIHC, LUAD, etc.	overexpressed	overexpressed in IDC	−1.02	1.06	1.56	4.24	high expression is a bad prognosis (RFS, OS and DMSF)
7	*EZR*	Ezrin	overexpressed in BRCA, CESC, LIHC, etc.	overexpressed	overexpressed in DCIS	1	1.63	1.16	4.58	high expression is a bad prognosis (RFS)
8	*CDK1*	Cyclin-dependent kinase 1	overexpressed in BLCA, BRCA, CESC, LIHC, LUAD, etc.	overexpressed	overexpressed in IDC	1.14	1.26	1.79	4.6	high expression is a bad prognosis (DMSF)
9	*CCT5*	Chaperonin-containing TCP1 subunit 5	overexpressed in BLCA, BRCA, CESC, LIHC, LUAD, etc.	overexpressed	overexpressed in IDC	1.03	1.18	1.53	5.31	high expression is a bad prognosis (RFS and DMSF)
10	*ASPM*	Abnormal spindle microtubule assembly	overexpressed in BLCA, BRCA, CESC, LIHC, LUAD, etc.	overexpressed	overexpressed in IDC	1.14	1.31	1.85	6.14	high expression is a bad prognosis (RFS, OS and DMSF)
11	*TOP2A*	DNA topoisomerase II alpha	overexpressed in BLCA, BRCA, CESC, LIHC, LUAD, etc.	overexpressed	overexpressed in IDC	1.07	1.23	3.85	6.52	high expression is a bad prognosis (RFS, OS and DMSF)
12	*ANLN*	Anillin actin-binding protein	overexpressed in BLCA, BRCA, CESC, LIHC, LUAD, etc.	overexpressed	overexpressed in IDC	1.07	1.22	2.57	6.67	high expression is a bad prognosis (RFS)
13	*PLK1*	Polo-like kinase 1	overexpressed in BLCA, BRCA, CESC, LIHC, LUAD, etc.	overexpressed	overexpressed in IDC	−1.02	1.08	1.61	6.81	high expression is a bad prognosis (RFS, OS and DMSF)
14	*CENPF*	Centromere protein F	overexpressed in BLCA, BRCA, CESC, LIHC, LUAD, etc.	overexpressed	overexpressed in IDC	1.18	1.05	2.71	6.84	high expression is a bad prognosis (RFS, OS and DMSF)
15	*CCNA2*	Cyclin A2	overexpressed in BLCA, BRCA, CESC, LIHC, LUAD, etc.	overexpressed	overexpressed in IDC	−1.14	1.12	1.88	7.08	high expression is a bad prognosis (RFS, OS and DMSF)
16	*KIF11*	Kinesin family member 11	overexpressed in BLCA, BRCA, CESC, LIHC, LUAD, etc.	overexpressed	overexpressed in IDC	−1.06	1.08	2.1	7.19	high expression is a bad prognosis (RFS, OS and DMSF)
17	*DTL*	Denticleless E3 ubiquitin protein ligase homolog	overexpressed in BLCA, BRCA, CESC, LIHC, LUAD, etc.	overexpressed	overexpressed in IDC	1.04	1.18	1.64	7.38	high expression is a bad prognosis (RFS, OS and DMSF)
18	*CCNB1*	Cyclin B1	overexpressed in BLCA, BRCA, CESC, LIHC, LUAD, etc.	overexpressed	overexpressed in IDC	1	1.14	1.57	7.5	high expression is a bad prognosis (RFS and OS)
19	*KIF23*	Kinesin family member 23	overexpressed in BLCA, BRCA, CESC, LIHC, LUAD, etc.	overexpressed	overexpressed in IDC	−1.03	−1.02	1.56	7.99	high expression is a bad prognosis (RFS, OS and DMSF)
20	*MKI67*	Marker Of Proliferation Ki-67	overexpressed in BLCA, BRCA, CESC, LIHC, LUAD, etc.	overexpressed	overexpressed in IDC	1.03	1.28	2.66	8.19	high expression is a bad prognosis (RFS, OS and DMSF)
21	*NUSAP1*	Nucleolar and spindle-associated protein 1	overexpressed in BLCA, BRCA, CESC, LIHC, LUAD, etc.	overexpressed	overexpressed in IDC	1.06	1.36	2.18	8.83	high expression is a bad prognosis (RFS, OS and DMSF)
22	*TPX2*	TPX2 microtubule nucleation factor	overexpressed in BLCA, BRCA, CESC, LIHC, LUAD, etc.	overexpressed	overexpressed in IDC	−1.08	1.17	1.91	9.04	high expression is a bad prognosis (RFS, OS and DMSF)
23	*FOXM1*	Forkhead box M1	overexpressed in BLCA, BRCA, CESC, LIHC, LUAD, etc.	overexpressed	overexpressed in IDC	−1.06	1.06	1.52	9.81	high expression is a bad prognosis (RFS, OS and DMSF)
24	*CCNB2*	Cyclin B2	overexpressed in BLCA, BRCA, CESC, LIHC, LUAD, etc.	overexpressed	overexpressed in IDC	−1.02	1.15	1.67	10.44	high expression is a bad prognosis (RFS, OS and DMSF)

**Table 2:** Top 4 clusters with their representative enriched terms (one per cluster) from cellular components enrichment analysis. “Count” is the number of genes in the user-provided lists with membership in the given ontology term. “%” is the percentage of all of the user-provided genes that are found in the given ontology term (only input genes with at least one ontology term annotation are included in the calculation). “Log10(P)” is the *p*-value in log base 10. “Log10(q)” is the multi-test adjusted *p*-value in log base 10.

GO	Description	Count	%	Log10(p)	Log10(q)	Genes
CORUM:310	Cell-cycle kinase complex CDC2	4	16.67	−5.54	−1.84	*CCNB1*, *CDK1*, *PCNA*, *CCNB2*, *CCNA2*, *DCAF13*, *DTL*
GO:0000940	Condensed chromosome outer kinetochore	3	12.5	−2.5	0	*CCNB1*, *CENPF*, *PLK1*, *KIF11*, *TPX2*, *CKAP2*, *ASPM*
GO:0072686	Mitotic spindle	8	33.33	−2.19	0	*CDK1*, *ECT2*, *KIF11*, *PLK1*, *KIF23*, *TPX2*, *NUSAP1*, *ASPM*, *EZR*, *CCT5*, *CKAP2*, *CCNB1*, *CENPF*
GO:0044297	Cell body	3	12.5	−1.34	0	*EZR*, *CCT5*, *TPX2*

**Table 3:** Top 5 clusters with their representative enriched terms (one per cluster) from pathway enrichment analysis. “Count” is the number of genes in the user-provided lists with membership in the given ontology term. “%” is the percentage of all of the user-provided genes that are found in the given ontology term (only input genes with at least one ontology term annotation are included in the calculation). “Log10(P)” is the *p*-value in log base 10. “Log10(q)” is the multi-test adjusted *p*-value in log base 10.

GO	Description	Count	%	Log10(p)	Log10(q)	Genes
R-HSA-156711	Polo-like-kinase-mediated events	5	20.83	−7.07	−2.84	*CCNB1*, *CENPF*, *FOXM1*, *PLK1*, *CCNB2*, *IGF1*, *TOP2A*, *TPX2*
R-HSA-69273	Cyclin-A/B1/B2-associated events during G2/M transition	6	25	−6.85	−2.84	*CCNA2*, *CCNB1*, *CDK1*, *FOXM1*, *PLK1*, *CCNB2*, *CENPF*, *PCNA*, *TOP2A*, *ANLN*, *IGF1*, *DTL*, *ECT2*, *TPX2*, *EDN3*, *KIF23*, *NUSAP1*, *MKI67*, *EZR*
M5893	HALLMARK MITOTIC SPINDLE	12	50	−6.12	−2.42	*CDK1*, *CENPF*, *ECT2*, *KIF11*, *PLK1*, *TOP2A*, *EZR*, *CCNB2*, *KIF23*, *TPX2*, *NUSAP1*, *ANLN*, *CCNA2*, *MKI67*, *CCNB1*, *CKAP2*, *ASPM*
WP2361	Gastric cancer network 1	4	16.67	−3.96	−0.94	*CENPF*, *ECT2*, *TOP2A*, *TPX2*
GO:0030866	Cortical actin cytoskeleton organization	3	12.5	−3.14	−0.4	*ECT2*, *EZR*, *ANLN*, *IGF1*, *CCT5*

**Table 4:** Top 5 clusters with their representative enriched terms (one per cluster) cellular functions enrichment analysis. “Count” is the number of genes in the user-provided lists with membership in the given ontology term. “%” is the percentage of all of the user-provided genes that are found in the given ontology term (only input genes with at least one ontology term annotation are included in the calculation). “Log10(P)” is the *p*-value in log base 10. “Log10(q)” is the multi-test adjusted *p*-value in log base 10.

GO	Description	Count	%	Log10(p)	Log10(q)	Genes
M00693	Cell-cycle-G2/M transition	4	16.67	−5.07	−1.7	*CCNA2*, *CCNB1*, *CDK1*, *CCNB2*, *TPX2*
GO:0019901	Protein kinase binding	9	37.5	−3.12	0	*CCNA2*, *CCNB1*, *FOXM1*, *KIF11*, *PCNA*, *PLK1*, *TOP2A*, *EZR*, *TPX2*
GO:0008022	Protein C-terminus binding	5	20.83	−2.71	0	*CENPF*, *MKI67*, *PCNA*, *TOP2A*, *EZR*
GO:0015631	Tubulin binding	8	33.33	−1.64	0	*CENPF*, *KIF11*, *PLK1*, *EZR*, *KIF23*, *CCT5*, *TPX2*, *NUSAP1*
GO:0050839	Cell-adhesion molecule binding	4	16.67	−1.45	0	*IGF1*, *EZR*, *CCNB2*, *ANLN*

**Table 5:** Top 30 genes with highest Spearman’s correlation coefficient (r ≥ 0.5) predicted to co-express with CKAP2. Genes with an asterisk are also present in the list of 24 mitotically relevant genes. The *q*-value is derived from Benjamini–Hochberg FDR correction procedure.

	Gene Symbol	Cytoband	Spearman’s Correlation	*p*-Value	*q*-Value
1	*DIAPH3*	13q21.2	0.739389854	1.27 × 10^−172^	2.54 × 10^−168^
2	*BORA*	13q21.33	0.731464545	3.78 × 10^−167^	3.78 × 10^−163^
3	*CKAP2L*	2q14.1	0.689512954	3.87 × 10^−141^	2.58 × 10^−137^
4	*BRCA2*	13q13.1	0.687867544	3.29 × 10^−140^	1.65 × 10^−136^
5	ASPM*	1q31.3	0.679105968	2.32 × 10^−135^	9.29 × 10^−132^
6	*KNL1*	15q15.1	0.674458017	7.39 × 10^−133^	2.47 × 10^−129^
7	*ARHGAP11A*	15q13.3	0.669955242	1.78 × 10^−130^	5.10 × 10^−127^
8	*BUB1*	2q13	0.66822039	1.44 × 10^−129^	3.60 × 10^−126^
9	*RFC3*	13q13.2	0.667922988	2.05 × 10^−129^	4.57 × 10^−126^
10	KIF11*	10q23.33	0.66342863	4.26 × 10^−127^	8.54 × 10^−124^
11	ECT2*	3q26.31	0.661963278	2.38 × 10^−126^	4.33 × 10^−123^
12	*SGO2*	2q33.1	0.661022354	7.14 × 10^−126^	1.19 × 10^−122^
13	MKI67*	10q26.2	0.660220461	1.82 × 10^−125^	2.80 × 10^−122^
14	*DLGAP5*	14q22.3	0.659368342	4.89 × 10^−125^	6.99 × 10^−122^
15	*BUB1B*	15q15.1	0.658250777	1.78 × 10^−124^	2.38 × 10^−121^
16	KIF23*	15q23	0.657140181	6.39 × 10^−124^	8.00 × 10^−121^
17	*KIF14*	1q32.1	0.65321807	5.59 × 10^−122^	6.59 × 10^−119^
18	ANLN*	7p14.2	0.651426238	4.22 × 10^−121^	4.70 × 10^−118^
19	*CIP2A*	3q13.13	0.65101644	6.69 × 10^−121^	7.05 × 10^−118^
20	*PRR11*	17q22	0.649728789	2.83 × 10^−120^	2.83 × 10^−117^
21	*SGO1*	3p24.3	0.646315057	1.25 × 10^−118^	1.19 × 10^−115^
22	*SKA3*	13q12.11	0.644899156	5.93 × 10^−118^	5.40 × 10^−115^
23	*TTK*	6q14.1	0.643244495	3.62 × 10^−117^	3.15 × 10^−114^
24	*RACGAP1*	12q13.12	0.642394775	9.13 × 10^−117^	7.62 × 10^−114^
25	*GAS2L3*	12q23.1	0.642202897	1.12 × 10^−116^	9.01 × 10^−114^
26	CCNA2*	4q27	0.640527147	6.90 × 10^−116^	5.31 × 10^−113^
27	*DEPDC1*	1p31.3	0.64047746	7.28 × 10^−116^	5.40 × 10^−113^
28	CENPF*	1q41	0.640325562	8.57 × 10^−116^	6.13 × 10^−113^
29	*KIF15*	3p21.31	0.638416389	6.67 × 10^−115^	4.60 × 10^−112^
30	*CENPE*	4q24	0.635192431	2.06 × 10^−113^	1.38 × 10^−110^

## Data Availability

The HTA data that support the findings of this study are available upon reasonable request from the corresponding author [C.D. and F.D.]. The data are not publicly available due to legal restrictions with respect to research participant privacy and consent.

## Supplementary Materials

The following supporting information can be downloaded at: https://www.mdpi.com/article/10.3390/cancers14153759/s1. Figure S1: Network of enriched terms (from the enrichment analysis of 24 mitotically relevant genes) (from Figure 1) colored by cluster ID, where nodes that share the same cluster ID are typically close to each other. Figure S2: Comparison of enrichment analysis of differentially expressed genes (DEGs) from every step of filtering (*p*-value, FC, and PRECOG filters) and background gene list using Metascape. Bar graph of enriched terms across input gene list, colored by *p*-values. Results showed that different GO terms were found after every step of filtering (including *p*-value, FC, and PRECOG filters) when compared to the background list. Therefore, this analysis indicates that there was no bias regarding the primary initial gene list. The DEGs found were primarily enriched in pathways, cellular components, and functions related to the chromosome segregation. Figure S3: Network of enriched terms (from the enrichment of highly co-expressed genes with *CKAP2*) (from Figure 5A) colored by cluster ID, where nodes that share the same cluster ID are typically close to each other. Figure S4: Representative images from Human Protein Atlas (https://www.proteinatlas.org) showing low (antibody HPA008410) (A), intermediate (antibody HPA027821) (B), and intermediate (antibody HPA008410) (C) stainings with CKAP2 positive cells in invasive ductal carcinoma (IDC) patients. Insets: Arrows indicate cells with positive CKAP2 staining. Scale bar: 200 µM. Figure S5: Original Western blot for the Figure 7D. Figure S6: Original Western blot for Figure 8A. Table S1: Sequence of primers used for RT-qPCR studies. Table S2: Gene ontology terms (retrieved from AmiGo) used to select mitotically relevant genes. Table S3: All genes with highest Spearman’s correlation coefficient (r ≥ 0.5) predicted to co-express with CKAP2. Genes with an asterisk (which comprise 16 genes) are also present in the list of 24 mitotically relevant genes.
