# Supplementary material for: Knockdown of CKAP2 Inhibits Proliferation, Migration, and Aggregate Formation in Aggressive Breast Cancer
Source: Cancers (Basel). 2022 Aug 2;14(15):3759. doi: 10.3390/cancers14153759 (PMC9367390; doi:10.3390/cancers14153759)
Supplement: Supplementary file 1 [file cancers-14-03759-s001.zip › cancers-1823581-supplementary.pdf]

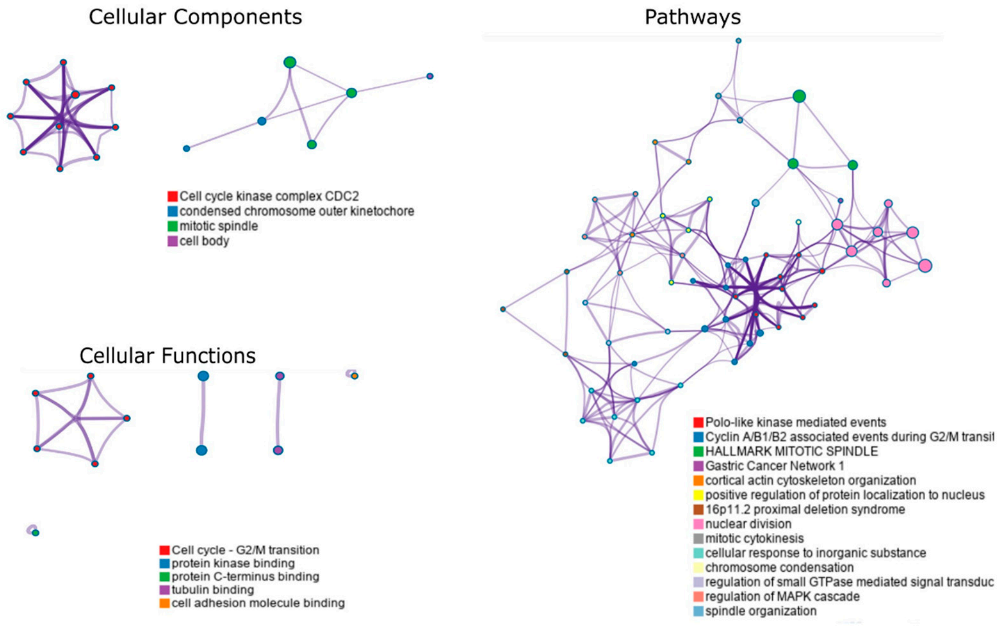

Figure S1. Network of enriched terms (from the enrichment analysis of 24 mitotically relevant genes) (from Figure 1) colored by cluster ID, where nodes that share the same cluster ID are typically close to each other.

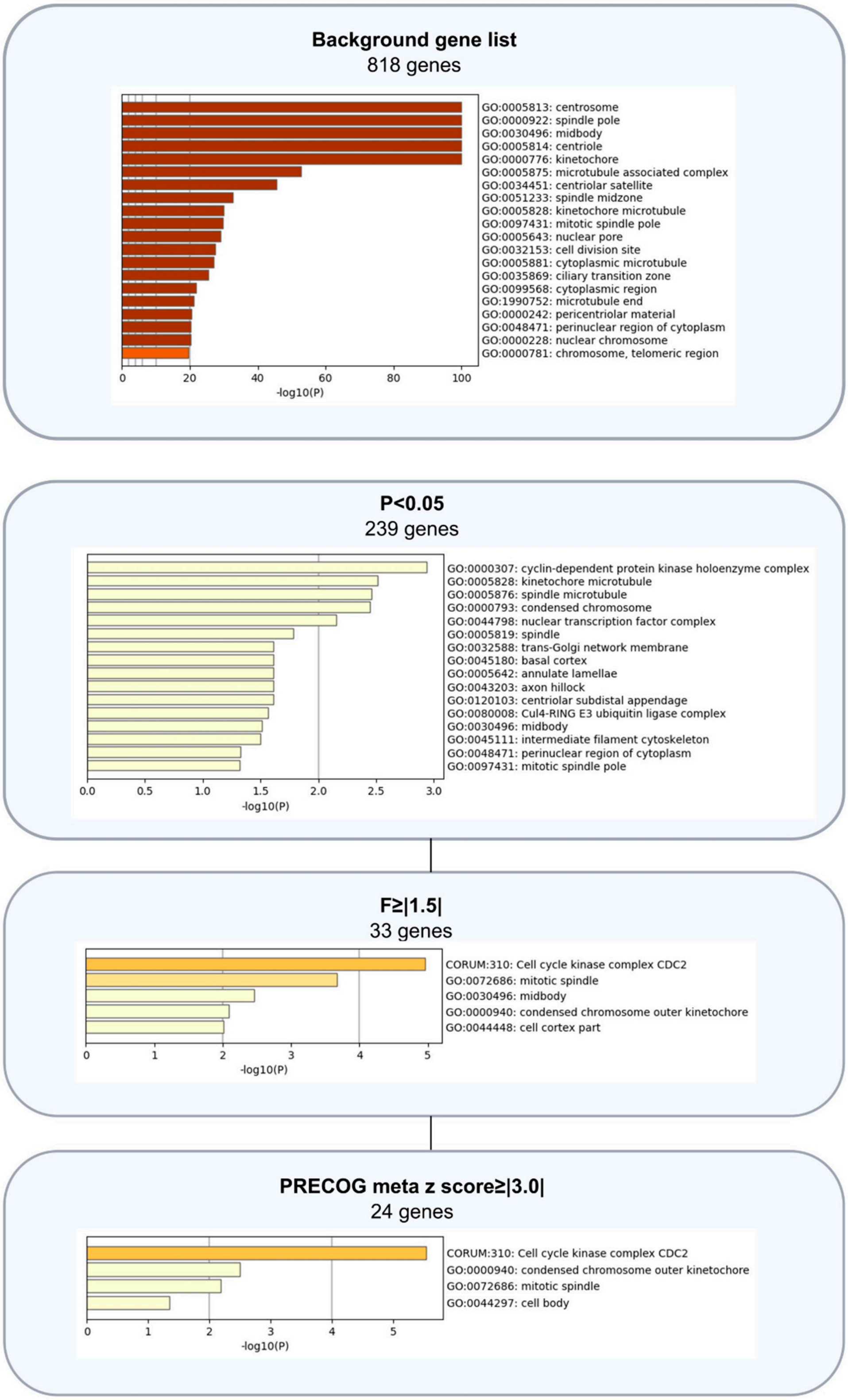

Figure S2. Comparison of enrichment analysis of differentially expressed genes (DEGs) from every step of filtering (p-value, FC, and PRECOG filters) and background gene list using Metascape. Bar graph of enriched terms across input gene list, colored by p-values. Results showed that different GO terms were found after every step of filtering (including p-value, FC, and PRECOG filters) when compared to the background list. Therefore, this analysis indicates that there was no bias regarding the primary initial gene list. And the DEGs found were primarily enriched in pathways, cellular components and functions related to the chromosome segregation.

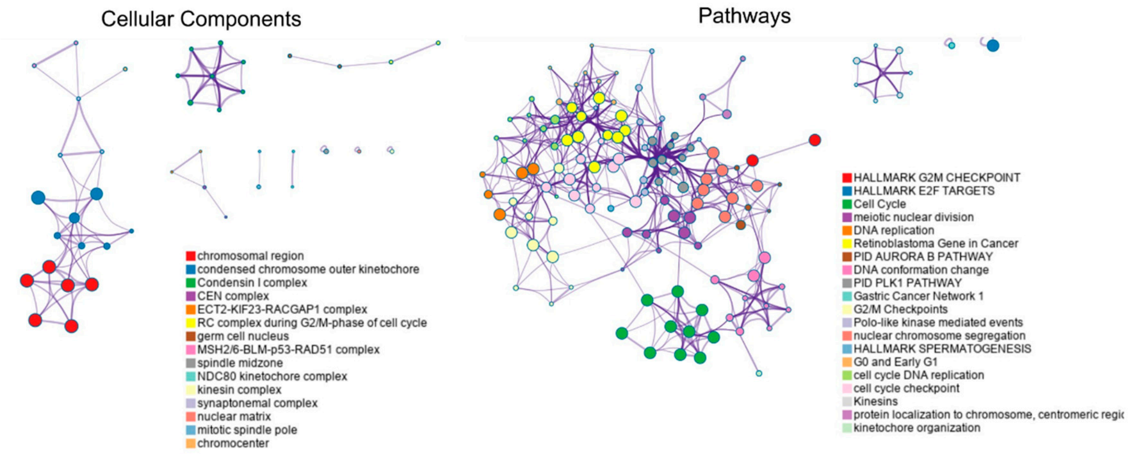

Figure S3. Network of enriched terms (from the enrichment of highly co-expressed genes with *CKAP2*) (from Figure 5A) colored by cluster ID, where nodes that share the same cluster ID are typically close to each other.

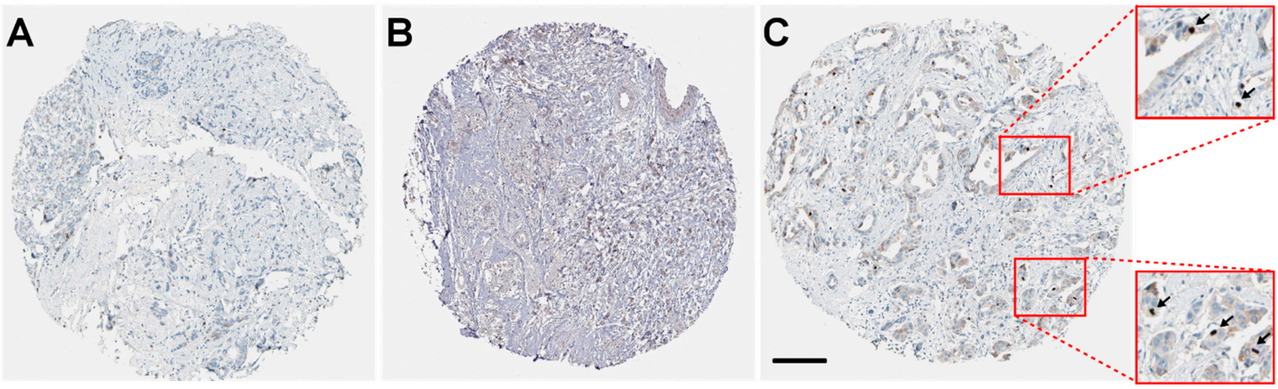

Figure S4. Representative images from Human Protein Atlas (www. https://www.proteinatlas.org) showing low (antibody HPA008410) (A), intermediate (antibody HPA027821) (B), and intermediate (antibody HPA008410) (C) stainings with CKAP2 positive cells in invasive ductal carcinoma (IDC) patients. Insets: Arrows indicate cells with positive CKAP2 staining. Scale bar: 200 μM.

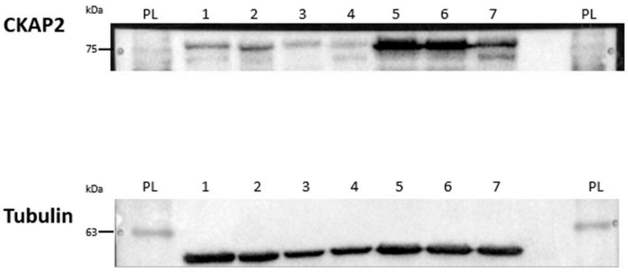

Figure S5. Original western blot for the Figure 7D. PL protein ladder; 1: MCF10A, 2: CA1; 3: MCF7 (luminal A); 4: BT474 (luminal B); 5: SKBR3 (HER2+); 6: MDA-MB-231 (TNBC); 7: MDA-MB-468 (TNBC); kDA: kilodaltons.

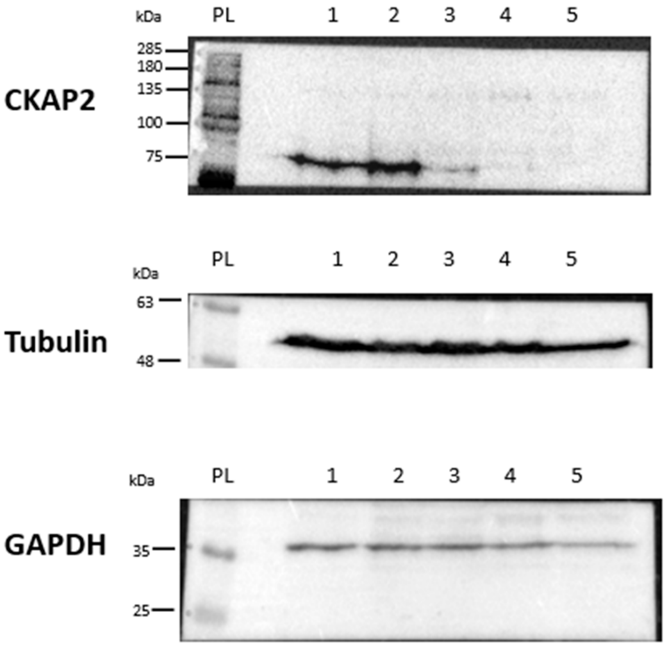

Figure S6. Original western blot for Figure 8A. PL: protein ladder; 1: parental cell line (non-transfected); 2: scrambled vector; 3: sh1; 4: sh2, 5: sh3; kDA: kilodaltons.

Table S1. Sequence of primers used for RT-qPCR studies.

Gene	Forward primer (5′-3′)	Reverse primer (5′-3′)
ASPM	TCAGAGATGGTTTCGAGCAAGA	AGCGAAATAGGGCTGCATCA
KIF11	C TGCCAGCAAG CTGCTTAAC	TGCATTGTGTTGGTCAACTGC
TPX2	CGGCTAATAACGGTTCTTGATACA	CAGGCCTACAGGTCTGACTC
CKAP2	CCGTTGACCAGCGAAGACATA	ACGAGCTTTTCTCTCTTCCGA
GAPDH	CGGGGCTCTCCAGAACATCAT	ATGCCAGTGAGCTTCCCGTTC
HPRT1	CGTCGTGATTAG-TGATGATGAACCA	ACACCCTTTCCAAATCCTCAGC

Table S2. Gene ontology terms (retrieved from AmiGo) used to select mitotically relevant genes.

Gene Ontology Identification (GO ID)	Description	Ontology
GO:0000022	mitotic spindle elongation	biological_process
GO:0000070	mitotic sister chromatid segregation	biological_process
GO:0000072	obsolete M phase specific microtubule process	biological_process
GO:0000073	spindle pole body separation	biological_process
GO:0000075	cell cycle checkpoint	biological_process
GO:0000078	obsolete cytokinesis after mitosis checkpoint	biological_process
GO:0000086	G2/M transition of mitotic cell cycle	biological_process
GO:0000087	mitotic M phase	biological_process
GO:0000088	mitotic prophase	biological_process
GO:0000089	mitotic metaphase	biological_process
GO:0000090	mitotic anaphase	biological_process
GO:0000091	mitotic anaphase A	biological_process
GO:0000092	mitotic anaphase B	biological_process
GO:0000093	mitotic telophase	biological_process

GO:0000117	regulation of transcription involved in G2/M transition of mitotic cell cycle	biological_process
GO:0000132	establishment of mitotic spindle orientation	biological_process
GO:0000159	protein phosphatase type 2A complex	cellular_component
GO:0000164	protein phosphatase type 1 complex	cellular_component
GO:0000212	meiotic spindle organization	biological_process
GO:0000216	obsolete M/G1 transition of mitotic cell cycle	biological_process
GO:0000226	microtubule cytoskeleton organization	biological_process
GO:0000231	obsolete cytoplasmic mitotic chromosome	cellular_component
GO:0000235	astral microtubule	cellular_component
GO:0000236	mitotic prometaphase	biological_process
GO:0000242	pericentriolar material	cellular_component
GO:0000278	mitotic cell cycle	biological_process
GO:0000279	M phase	biological_process
GO:0000280	nuclear division	biological_process
GO:0000281	mitotic cytokinesis	biological_process
GO:0000320	re-entry into mitotic cell cycle	biological_process
GO:0000751	mitotic cell cycle G1 arrest in response to pheromone	biological_process
GO:0000775	chromosome, centromeric region	cellular_component
GO:0000776	kinetochore	cellular_component
GO:0000777	condensed chromosome kinetochore	cellular_component
GO:0000778	condensed nuclear chromosome kinetochore	cellular_component
GO:0000779	condensed chromosome, centromeric region	cellular_component
GO:0000780	condensed nuclear chromosome, centromeric region	cellular_component
GO:0000818	nuclear MIS12/MIND complex	cellular_component
GO:0000819	sister chromatid segregation	biological_process
GO:0000910	cytokinesis	biological_process
GO:0000911	cytokinesis by cell plate formation	biological_process
GO:0000912	assembly of actomyosin apparatus involved in cytokinesis	biological_process
GO:0000915	actomyosin contractile ring assembly	biological_process
GO:0000916	actomyosin contractile ring contraction	biological_process
GO:0000917	barrier septum assembly	biological_process
GO:0000918	barrier septum site selection	biological_process
GO:0000919	cell plate assembly	biological_process
GO:0000920	cell separation after cytokinesis	biological_process
GO:0000921	septin ring assembly	biological_process
GO:0000922	spindle pole	cellular_component
GO:0000923	equatorial microtubule organizing center	cellular_component
GO:0000924	gamma-tubulin ring complex, centrosomal	cellular_component
GO:0000927	gamma-tubulin small complex, centrosomal	cellular_component
GO:0000928	gamma-tubulin small complex, spindle pole body	cellular_component
GO:0000930	gamma-tubulin complex	cellular_component
GO:0000930	gamma-tubulin complex	cellular_component
GO:0000931	gamma-tubulin large complex	cellular_component
GO:0000940	condensed chromosome outer kinetochore	cellular_component
GO:0000941	condensed nuclear chromosome inner kinetochore	cellular_component
GO:0000942	condensed nuclear chromosome outer kinetochore	cellular_component
GO:0001100	negative regulation of exit from mitosis	biological_process
GO:0001578	microtubule bundle formation	biological_process
GO:0003425	establishment of mitotic spindle orientation involved in growth plate cartilage chondrocyte division	biological_process

Table S3. All genes with highest Spearman's correlation coefficient (r ≥ 0.5) predicted to co-express with *CKAP2*. Genes with an asterisk (which comprise 16 genes) are also present in the 24 mitotically relevant gene list. The q-value is derived from Benjamini-Hochberg FDR correction procedure.

	Gene Symbol	Cytoband	Spearman's Correlation	p-Value	q-Value
1	*DIAPH3*	13q21.2	0.739389854	1.27E-172	2.54E-168
2	*BORA*	13q21.33	0.731464545	3.78E-167	3.78E-163
3	*CKAP2L*	2q14.1	0.689512954	3.87E-141	2.58E-137
4	*BRCA2*	13q13.1	0.687867544	3.29E-140	1.65E-136
5	*ASPM**	1q31.3	0.679105968	2.32E-135	9.29E-132
6	*KNL1*	15q15.1	0.674458017	7.39E-133	2.47E-129
7	*ARHGAP11A*	15q13.3	0.669955242	1.78E-130	5.10E-127
8	*BUB1*	2q13	0.66822039	1.44E-129	3.60E-126
9	*RFC3*	13q13.2	0.667922988	2.05E-129	4.57E-126
10	*KIF11**	10q23.33	0.66342863	4.26E-127	8.54E-124
11	*ECT2**	3q26.31	0.661963278	2.38E-126	4.33E-123
12	*SGO2*	2q33.1	0.661022354	7.14E-126	1.19E-122
13	*MKI67**	10q26.2	0.660220461	1.82E-125	2.80E-122
14	*DLGAP5*	14q22.3	0.659368342	4.89E-125	6.99E-122
15	*BUB1B*	15q15.1	0.658250777	1.78E-124	2.38E-121
16	*KIF23**	15q23	0.657140181	6.39E-124	8.00E-121
17	*KIF14*	1q32.1	0.65321807	5.59E-122	6.59E-119
18	*ANLN**	7p14.2	0.651426238	4.22E-121	4.70E-118
19	*CIP2A*	3q13.13	0.65101644	6.69E-121	7.05E-118
20	*PRR11*	17q22	0.649728789	2.83E-120	2.83E-117
21	*SGO1*	3p24.3	0.646315057	1.25E-118	1.19E-115
22	*SKA3*	13q12.11	0.644899156	5.93E-118	5.40E-115
23	*TTK*	6q14.1	0.643244495	3.62E-117	3.15E-114
24	*RACGAP1*	12q13.12	0.642394775	9.13E-117	7.62E-114
25	*GAS2L3*	12q23.1	0.642202897	1.12E-116	9.01E-114
26	*CCNA2**	4q27	0.640527147	6.90E-116	5.31E-113
27	*DEPDC1*	1p31.3	0.64047746	7.28E-116	5.40E-113
28	*CENPF**	1q41	0.640325562	8.57E-116	6.13E-113
29	*KIF15*	3p21.31	0.638416389	6.67E-115	4.60E-112
30	*CENPE*	4q24	0.635192431	2.06E-113	1.38E-110
31	*KIF20B*	10q23.31	0.635139865	2.18E-113	1.41E-110
32	*STIL*	1p33	0.633722239	9.72E-113	6.08E-110
33	*CENPI*	Xq22.1	0.632441761	3.73E-112	2.26E-109
34	*HMMR*	5q34	0.631166892	1.41E-111	8.31E-109
35	*SMC2*	9q31.1	0.629679349	6.62E-111	3.79E-108
36	*NUSAP1**	15q14	0.629128983	1.17E-110	6.52E-108
37	*KIF18A*	11p14.1	0.623139608	5.37E-108	2.91E-105
38	*PLK4*	4q28.1	0.621343946	3.29E-107	1.73E-104
39	*ARHGAP11B*	15q13.2	0.620381771	8.64E-107	4.44E-104
40	*CEP55*	10q23.33	0.620097211	1.15E-106	5.75E-104
41	*TOP2A**	17q21.2	0.618861309	3.95E-106	1.93E-103
42	*FAM83D*	20q11.23	0.618378557	6.39E-106	3.05E-103
43	*FBXO5*	6q25.2	0.618157622	7.95E-106	3.71E-103
44	*HASPIN*	17p13.2	0.614756758	2.29E-104	1.04E-101
45	*KIF20A*	5q31.2	0.614647071	2.55E-104	1.14E-101
46	*POLQ*	3q13.33	0.614622911	2.61E-104	1.14E-101
47	*NCAPH*	2q11.2	0.611148413	7.74E-103	3.30E-100
48	*RAD51AP1*	12p13.32	0.609585426	3.51E-102	1.46E-99

49	*CLSPN*	1p34.3	0.609426832	4.09E-102	1.67E-99
50	*ERCC6L*	Xq13.1	0.608591958	9.12E-102	3.65E-99
51	*PROSER1*	13q13.3	0.608276456	1.23E-101	4.85E-99
52	*NCAPG*	4p15.31	0.607953978	1.68E-101	6.48E-99
53	*PARPBP*	12q23.2	0.605995083	1.09E-100	4.13E-98
54	*XPO1*	2p15	0.60593109	1.16E-100	4.31E-98
55	*TPX2**	20q11.21	0.605908644	1.19E-100	4.32E-98
56	*SMC4*	3q25.33	0.605632106	1.54E-100	5.52E-98
57	*TMPO*	12q23.1	0.605161846	2.41E-100	8.47E-98
58	*SKP2*	5p13.2	0.604133634	6.38E-100	2.20E-97
59	*DEPDC1B*	5q12.1	0.601143896	1.06E-98	3.59E-96
60	*KPNA3*	13q14.2	0.60113141	1.07E-98	3.59E-96
61	*E2F8*	11p15.1	0.599066399	7.36E-98	2.42E-95
62	*MELK*	9p13.2	0.59884826	9.02E-98	2.91E-95
63	*NEIL3*	4q34.3	0.597597988	2.87E-97	9.12E-95
64	*E2F7*	12q21.2	0.596425358	8.46E-97	2.65E-94
65	*MCM10*	10p13	0.595803438	1.50E-96	4.62E-94
66	*KIF4A*	Xq13.1	0.594215531	6.41E-96	1.94E-93
67	*OBI1*	13q31.1	0.593513869	1.21E-95	3.63E-93
68	*MAD2L1*	4q27	0.590758337	1.48E-94	4.35E-92
69	*NCAPD2*	12p13.31	0.586686765	5.66E-93	1.64E-90
70	*PRC1*	15q26.1	0.585499772	1.62E-92	4.64E-90
71	*CDCA2*	8p21.2	0.584605136	3.58E-92	1.01E-89
72	*NDC80*	18p11.32	0.584152403	5.33E-92	1.48E-89
73	*FAM72A*	1q32.1	0.581835733	4.07E-91	1.12E-88
74	*BLM*	15q26.1	0.581360096	6.17E-91	1.67E-88
75	*LMNB1*	5q23.2	0.580766813	1.04E-90	2.77E-88
76	*HJURP*	2q37.1	0.580501216	1.30E-90	3.44E-88
77	*SPDL1*	5q35.1	0.57988857	2.22E-90	5.78E-88
78	*CDK1**	10q21.2	0.57975559	2.49E-90	6.41E-88
79	*MSH2*	2p21-p16.3	0.579332123	3.60E-90	9.13E-88
80	*TFDP1*	13q34	0.577764232	1.40E-89	3.50E-87
81	*CCNB2**	15q22.2	0.577069174	2.54E-89	6.28E-87
82	*ZNF367*	9q22.32	0.57653025	4.04E-89	9.86E-87
83	*GEN1*	2p24.2	0.576482891	4.21E-89	1.01E-86
84	*WDHD1*	14q22.2-q22.3	0.575512545	9.66E-89	2.30E-86
85	*ESCO2*	8p21.1	0.573885031	3.87E-88	9.12E-86
86	*GPR180*	13q32.1	0.571960762	1.98E-87	4.61E-85
87	*FOXM1**	12p13.33	0.570430803	7.18E-87	1.65E-84
88	*GPSM2*	1p13.3	0.567234154	1.04E-85	2.37E-83
89	*FANCD2*	3p25.3	0.567150825	1.11E-85	2.51E-83
90	*CDCA8*	1p34.3	0.563824086	1.74E-84	3.88E-82
91	*NCAPG2*	7q36.3	0.56201332	7.68E-84	1.69E-81
92	*CIT*	12q24.23	0.561810426	9.06E-84	1.95E-81
93	*GTSE1*	22q13.31	0.561805491	9.10E-84	1.95E-81
94	*FANCB*	Xp22.2	0.561798668	9.15E-84	1.95E-81
95	*CDK8*	13q12.13	0.560414734	2.82E-83	5.95E-81
96	*ATAD2*	8q24.13	0.559943309	4.14E-83	8.63E-81
97	*CENPO*	2p23.3	0.559565285	5.62E-83	1.16E-80
98	*NUF2*	1q23.3	0.559465221	6.09E-83	1.25E-80
99	*DBF4*	7q21.12	0.557288942	3.53E-82	7.14E-80
100	*KIF4B*	5q33.2	0.556219785	8.31E-82	1.67E-79

101	*SKA1*	18q21.1	0.555334833	1.69E-81	3.35E-79
102	*CENPJ*	13q12.12-q12.13	0.554848172	2.49E-81	4.88E-79
103	*TICRR*	15q26.1	0.554714678	2.77E-81	5.38E-79
104	*CENPA*	2p23.3	0.554555651	3.14E-81	6.05E-79
105	*CCNB1**	5q13.2	0.553924469	5.19E-81	9.90E-79
106	*RAP2A*	13q32.1	0.551021598	5.15E-80	9.73E-78
107	*DNA2*	10q21.3	0.54983991	1.30E-79	2.44E-77
108	*KPNA2*	17q24.2	0.549533989	1.65E-79	3.07E-77
109	*MZT1*	13q21.33	0.549015699	2.48E-79	4.56E-77
110	*NEK2*	1q32.3	0.548945138	2.62E-79	4.78E-77
111	*AURKA*	20q13.2	0.548327397	4.25E-79	7.66E-77
112	*INCENP*	11q12.3	0.547638476	7.26E-79	1.30E-76
113	*FAM72B*	1p11.2	0.546989528	1.20E-78	2.13E-76
114	*EXO1*	1q43	0.546866004	1.32E-78	2.33E-76
115	*SUV39H2*	10p13	0.545962149	2.66E-78	4.64E-76
116	*CCNE2*	8q22.1	0.544474196	8.40E-78	1.45E-75
117	*PLK1**	16p12.2	0.54420365	1.03E-77	1.77E-75
118	*FANCI*	15q26.1	0.543443703	1.85E-77	3.15E-75
119	*MIS18BP1*	14q21.2	0.543065141	2.48E-77	4.17E-75
120	*TOPBP1*	3q22.1	0.540631348	1.59E-76	2.65E-74
121	*FAM72D*	1q21.1	0.540580885	1.65E-76	2.73E-74
122	*KIF2C*	1p34.1	0.54052975	1.71E-76	2.81E-74
123	*RBL1*	20q11.23	0.540334399	1.99E-76	3.24E-74
124	*ZWILCH*	15q22.31	0.539724819	3.15E-76	5.09E-74
125	*NUDT15*	13q14.2	0.539159859	4.84E-76	7.75E-74
126	*PRIM2*	6p11.2	0.537959612	1.20E-75	1.90E-73
127	*SRPK1*	6p21.31	0.537734726	1.42E-75	2.23E-73
128	*SHCBP1*	16q11.2	0.536060786	4.97E-75	7.78E-73
129	*FAM111B*	11q12.1	0.534548352	1.54E-74	2.39E-72
130	*CDC25A*	3p21.31	0.532677936	6.16E-74	9.49E-72
131	*NEMP1*	12q13.3	0.530795413	2.47E-73	3.77E-71
132	*ORC1*	1p32.3	0.528799877	1.06E-72	1.61E-70
133	*CKAP5*	11p11.2	0.528088021	1.79E-72	2.69E-70
134	*RTKN2*	10q21.2	0.527653305	2.45E-72	3.67E-70
135	*RFWD3*	16q23.1	0.527327572	3.11E-72	4.61E-70
136	*TRIP13*	5p15.33	0.527256078	3.27E-72	4.82E-70
137	*MCM4*	8q11.21	0.526968526	4.03E-72	5.90E-70
138	*ESPL1*	12q13.13	0.52695666	4.07E-72	5.91E-70
139	*BRIP1*	17q23.2	0.524039635	3.34E-71	4.82E-69
140	*SPC25*	2q31.1	0.52400746	3.42E-71	4.90E-69
141	*TTF2*	1p13.1	0.522701328	8.73E-71	1.24E-68
142	*ATAD5*	17q11.2	0.520333009	4.72E-70	6.66E-68
143	*CEP152*	15q21.1	0.519810768	6.83E-70	9.57E-68
144	*MSH6*	2p16.3	0.518259575	2.05E-69	2.83E-67
145	*MTBP*	8q24.12	0.517085901	4.67E-69	6.41E-67
146	*CENPN*	16q23.2	0.515863685	1.10E-68	1.50E-66
147	*IPO5*	13q32.2	0.515793854	1.15E-68	1.56E-66
148	*FIGNL1*	7p12.2	0.515141344	1.82E-68	2.45E-66
149	*PBK*	8p21.1	0.514318772	3.23E-68	4.30E-66
150	*DSCC1*	8q24.12	0.514312125	3.24E-68	4.30E-66
151	*CENPL*	1q25.1	0.511570814	2.17E-67	2.85E-65
152	*MTFR2*	6q23.3	0.510972342	3.27E-67	4.28E-65

153	*TUBGCP3*	13q34	0.510923528	3.38E-67	4.40E-65
154	*IQGAP3*	1q22	0.50983203	7.15E-67	9.25E-65
155	*HELLS*	10q23.33	0.508473582	1.81E-66	2.33E-64
156	*MCM8*	20p12.3	0.507948467	2.59E-66	3.28E-64
157	*SMC1A*	Xp11.22	0.505622081	1.26E-65	1.58E-63
158	*SPAG5*	17q11.2	0.50554846	1.32E-65	1.65E-63
159	*GINS1*	20p11.21	0.505097396	1.79E-65	2.23E-63
160	*DDIAS*	11q14.1	0.504948741	1.98E-65	2.45E-63
161	*XRCC2*	7q36.1	0.50390581	3.99E-65	4.91E-63
162	*XPO5*	6p21.1	0.502598994	9.59E-65	1.17E-62
163	*LIN9*	1q42.12	0.502530167	1.00E-64	1.22E-62
164	*NUP205*	7q33	0.501012747	2.76E-64	3.33E-62
